# Abraxas suppresses DNA end resection and limits break-induced replication by controlling SLX4/MUS81 chromatin loading in response to TOP1 inhibitor-induced DNA damage

**DOI:** 10.1038/s41467-021-24665-w

**Published:** 2021-07-16

**Authors:** Xiao Wu, Bin Wang

**Affiliations:** 1 Department of Genetics, The University of Texas MD Anderson Cancer Center, Houston, Texas USA; 2 Genetics and Epigenetics Program, The MD Anderson Cancer Center UT Health Graduate School of Biomedical Sciences, Houston, Texas USA

**Keywords:** Double-strand DNA breaks, Ubiquitylation

## Abstract

Although homologous recombination (HR) is indicated as a high-fidelity repair mechanism, break-induced replication (BIR), a subtype of HR, is a mutagenic mechanism that leads to chromosome rearrangements. It remains poorly understood how cells suppress mutagenic BIR. Trapping of Topoisomerase 1 by camptothecin (CPT) in a cleavage complex on the DNA can be transformed into single-ended double-strand breaks (seDSBs) upon DNA replication or colliding with transcriptional machinery. Here, we demonstrate a role of Abraxas in limiting seDSBs undergoing BIR-dependent mitotic DNA synthesis. Through counteracting K63-linked ubiquitin modification, Abraxas restricts SLX4/Mus81 recruitment to CPT damage sites for cleavage and subsequent resection processed by MRE11 endonuclease, CtIP, and DNA2/BLM. Uncontrolled SLX4/MUS81 loading and excessive end resection due to Abraxas-deficiency leads to increased mitotic DNA synthesis via RAD52- and POLD3- dependent, RAD51-independent BIR and extensive chromosome aberrations. Our work implicates Abraxas/BRCA1-A complex as a critical regulator that restrains BIR for protection of genome stability.

## Abstract (web summary)

Break-induced replication (BIR), a subtype of HR, is a mutagenic mechanism that leads to chromosome rearrangements. Here the authors reveal insights into the role of Abraxas in limiting excessive DNA end resection, R-loop accumulation and cells undergoing BIR-dependent mitotic DNA synthesis.

## Introduction

Topoisomerase I (TOP1) relaxes topological stress generated during DNA replication and transcription by temporarily forming a reversible TOP1-DNA cleavage complex (TOP1cc) nicking one strand and allowing rotation of the broken strand around the intact strand^1,2^. TOP1 inhibitor camptothecin (CPT) and its clinical derivatives traps TOP1ccs at the enzyme–DNA interface forming protein–DNA crosslinks blocking DNA replication fork progression and ongoing transcription^3^. Replication forks colliding with the stabilized TOP1ccs induce replication-associated DNA double-strand breaks (DSBs). These are single-ended DSBs (seDSBs) caused by either replication run-off or cleavage of stalled replication forks by MUS81^4^. Poisoning of TOP1 by CPT also induces accumulation of unscheduled R-loop, a three-stranded structure formed during transcription, which contain DNA–RNA hybrids and single-stranded displaced non-template DNA^5–10^. R loops induced by stalled Top1ccs lead to generation of transcription-dependent DSBs^11^. R-loop-impeded replication forks can be sensed and cleaved by MUS81 leading to DSBs^12^.

Two major pathways, homologous recombination (HR) and nonhomologous end joining (NHEJ) are involved in DSBs repair^13^. HR begins through DNA end resection that is initiated by MRE11-RAD50-NBS1 complex and CtIP through MRE11 endonuclease activity creating an adjacent DNA nick followed by nucleolytic processes by EXO1, DNA2 nucleases, and MRE11 exonuclease activity in both directions from the nick^14^. Compared to two-ended DSBs, seDSBs lack another DNA end to be ligated to for end joining, and thus are preferentially repaired by HR^13^. In budding yeast, it has been established that seDSBs repair undergo break-induced replication (BIR), a type of HR process that involves extensive 5′ to 3′-end resection to generate a 3′-single-stranded DNA (ssDNA) end that is bound by RPA, RAD52-mediated strand invasion and formation of displacement loop (D-loop) intermediates and POLD3-dependent DNA synthesis^15–17^. In human cells, MUS81-mediated cleavage of collapsed replication forks at common fragile sites (CFS) initiates mitotic DNA synthesis (MiDAS) via BIR mechanism that depends on RAD52 and POLD3 but not on RAD51^18,19^. BIR also occurs in response to oncogene overexpression, re-replication, or telomere erosion^17,20^. Although HR is indicated as a high-fidelity repair mechanism, BIR is a mutagenic mechanism that leads to chromosome rearrangements. The mechanism of resection to generate 3′-ssDNA and regulation of BIR has been extensively studied in yeast but still remains poorly understood in mammalian cells.

Abraxas interacts with BRCA1, forming a BRCA1-A complex with additional components Rap80, BRE, NBA1, and BRCC36^21^. Abraxas is the central adapter protein of the BRCA1-A complex mediating the interaction of BRCA1 with the rest of the components^21–25^. Abraxas/BRCA1-A complex is recruited to DSBs in an Ataxia-Telangiectasia Mutated (ATM)-dependent signaling cascade that involves ubiquitin lysine 63 (K63)-linked modification on damaged chromatin catalyzed by E3 ligases RNF8/RNF168 and a K63-linkage-specific E2 conjugating enzyme UBC13^26,27^. It has been shown that Abraxas and the formation of the BRCA1-A complex facilitates the deubiquitinating (DUB) activity of BRCC36 to disassemble K63-linked ubiquitin chain^28,29^. Despite the functional importance of Abraxas in recruitment of BRCA1 and tumor suppression^21–24,30,31^, the role of Abraxas in DNA repair still needs to be defined. In addition, the functional importance of Abraxas/BRCA1-A complex as a DUB complex is not clear.

BRCA1-A complex have been indicated a role in suppressing DSB resection, as defects in Rap80 and several other components of the BRCA1-A complex are linked to increased resection and appear to increase HR efficiency using a reporter assay^32–34^. It is intriguing how the Abraxas/BRCA1-A complex suppresses DNA end resection and HR, while BRCA1 plays a promoting role in facilitating DNA end resection and HR.

Here we found that Abraxas inhibits excessive DNA end resection at CPT-induced seDSB sites by regulating K63-linked ubiquitin modification-dependent SLX4/MUS81 chromatin loading and subsequent cleavage of replication-associated DSBs. In the absence of Abraxas, increased K63-linked polyubiquitination led to enrichment of SLX4/MUS81 on damaged chromatin, causing excessive cleavage of CPT-induced stalled replication forks and R loops. DNA ends of cleaved replication-associated DSBs are further processed by MRE11 endonuclease, CTIP, and DNA2/BLM nucleases, generating excessive ssDNA that leads to MiDAS via BIR mechanism and chromosome aberrations. Our results demonstrate a role of Abraxas in regulating replication-associated seDSBs repair through inhibiting DNA synthesis-dependent BIR to safeguard genome stability.

## Results

### Abraxas limits DNA end resection of replication-associated DSBs

**Fig. 1:**
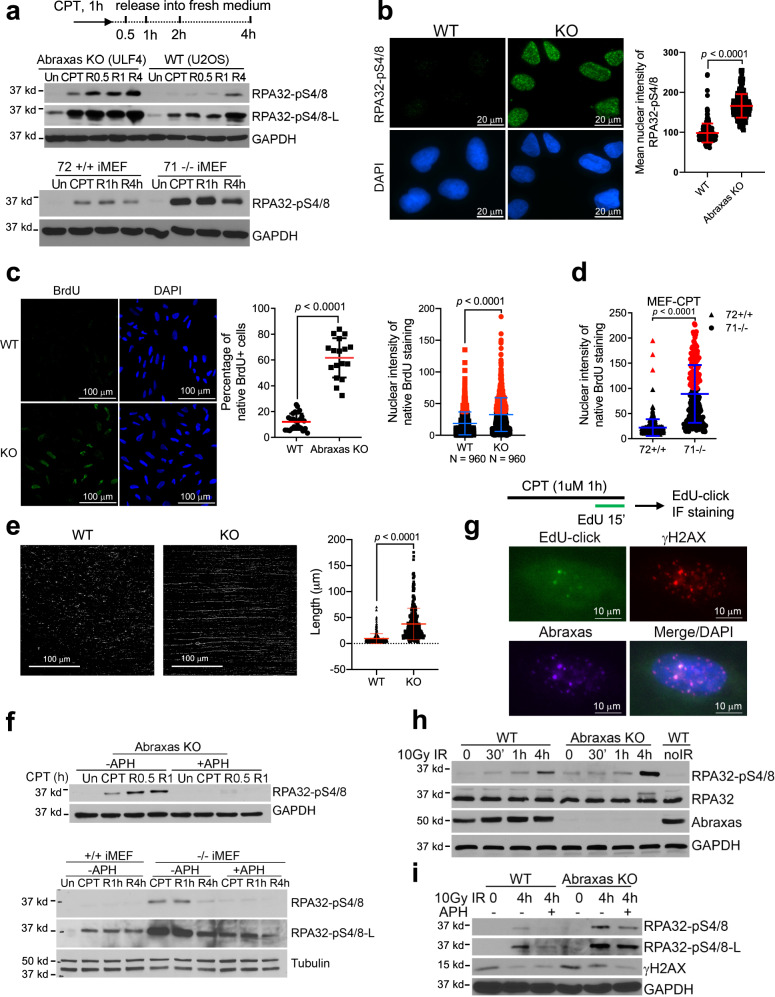
Abraxas limits DNA end resection of replication-associated DSBs. **a** Increased RPA32-pS4/8 levels in *Abraxas* knockout (KO) U2OS and *Abraxas−/−* MEF cells in response to CPT. Cells were untreated (Un), treated with 1 μM CPT for 1 h, released into fresh medium, and collected at indicated times (R0.5, R1, R4). **b** Immunofluorescence staining of RPA32-pS4/8 in WT and *Abraxas* KO U2OS cells treated with 1 μM CPT for 1 h. Cells were pre-extracted with 0.2 % Triton X-100 before fixation. Nuclear intensity of RPA32-pS4/8 was quantified by ImageJ, shown as mean value ± SD for WT (*n* = 244), KO (*n* = 187) cells examined. Two-tailed unpaired *t*-test was used for statistical analysis. **c** Increased ssDNA accumulation in *Abraxas* KO U2OS cells with CPT treatment as detected by native BrdU immunofluorescence staining. Cells were labeled with BrdU for 36 h before being treated with 1 μM CPT for 1 h. Percentage of BrdU+ cells were plotted as mean value ± SD for WT (*n* = 24 independent image areas with total 960 cells), KO (*n* = 17 independent image areas with total 960 cells). BrdU nuclear intensity were plotted as mean value ± SD for WT (*n* = 960), KO (*n* = 960) cells examined. Two-tailed unpaired *t*-test was used for statistical analysis. Red dots represent BrdU nuclear intensity values higher than 40. **d** Increased ssDNA accumulation in *Abraxas−/−* MEF cells in response to CPT (1 μM, 1 h) detected by native BrdU immunofluorescence staining. BrdU intensity was measured using ImageJ and plotted as mean value ± SD for *Abraxas*+*/+* (72+/+, *n* = 382) or *−/−* (71−/−, *n* = 216) cells examined. Two-tailed unpaired *t*-test was used for statistical analysis. Red dots represent BrdU nuclear intensity values higher than 100. **e** Increased resection in *Abraxas* KO cells treated with CPT. Cells labeled with BrdU for 24 h were treated with CPT (1 μM, 1 h). DNA fibers were stained with BrdU under native condition. Total fibers were also stained with YOYO-1 and shown in Supplementary Fig. 1c. The length of BrdU-stained DNA fiber was measured by ImageJ and plotted as mean value ± SD for WT (*n* = 246) and KO (*n* = 371) DNA fibers. Two-tailed unpaired *t*-test was used for statistical analysis. **f** Inhibition of DNA replication by aphidicolin (APH) abolished RPA hyperphosphorylation in Abraxas-deficient cells. APH (0.1 μM) was added to medium 15 min before cells were treated with 1 μM CPT. Cells were then released into fresh medium containing APH and collected at indicated times. **g** Abraxas accumulates at replication-associated DSBs. Cells were treated with CPT (1 μM, 1 h), pulsed labeled with EdU for 15 min before EdU-click and IF staining with antibodies to Abraxas and γH2AX. **h** Elevated RPA32-pS4/8 levels in *Abraxas* KO cells at 4 h after IR treatment. WT and KO cells were treated with 10 Gy IR and collected at 0, 30 min, 1 h, and 4 h. Western blottings were performed using indicated antibodies. **i** Inhibition of replication by APH reduced RPA hyperphosphorylation upon 10 Gy IR. APH was added to medium 15 min before IR. RPA32-pS4/8 levels were compared between WT and Abraxas KO cells at 4 h after 10 Gy IR without or with 0.1 μM APH incubation. Student’s *t*-test was used for statistical analysis of **b**–**d**. See also Supplementary Fig. 1.

We found that when treated with CPT, *Abraxas* knockout (KO) U2OS cells or *Abraxas* null *(−/−)* mouse embryonic fibroblast (MEF) cells displayed increased phosphorylation of RPA32 S4/8 (pRPA), a surrogate marker of ssDNA accumulation and DNA end resection, upon treatment and at the indicated times after release into fresh media when compared to the control cells (Fig. 1a and Supplementary Fig. 1a). Total RPA32 level was not changed in Abraxas-deficient cells (Supplementary Fig. 1b). Immunofluorescence staining (IF) also showed increased staining of pRPA in *Abraxas* KO cells upon CPT treatment (Fig. 1b). Importantly, complementation with expression of hemagglutinin (HA)-tagged *Abraxas* in KO cells reduced the increased pRPA levels (Supplementary Fig. 1b). The increased pRPA level is correlated with increased ssDNA in *Abraxas* KO cells and *Abraxas−/−* MEFs detected by native Bromodeoxyuridine (BrdU) labeling and detection (Fig. 1c, d). To monitor DNA end resection, we carried out single-molecule analysis of resection tracks (SMART) to directly visualize resection at breaks^35^. It is apparent that *Abraxas* KO cells showed much increased resected BrdU-labeled ssDNA track length in response to CPT damage (Fig. 1e and Supplementary Fig. 1c). These data indicate that, compared to the control, there is increased DNA end resection and ssDNA generation in Abraxas-deficient cells.

CPT-induced stabilized Top1ccs can readily be converted into seDSBs as replication forks collide with the stabilized Top1ccs^4^. To determine whether increased resection in Abraxas-deficient cells is associated with replication, we used aphidicolin (APH), an inhibitor of DNA polymerase to inhibit DNA synthesis before the treatment of cells with CPT. We found that inhibition of replication by APH completely abolished the increased pRPA levels in response to CPT in *Abraxas* KO U2OS cells and *Abraxas*-null MEFs, indicating that Abraxas inhibits DNA end resection at collapsed replication forks (Fig. 1f).

We then examined whether Abraxas localizes to CPT-induced DNA damage sites. Upon CPT treatment, Abraxas forms CPT-induced DNA damage foci using IF (Fig. 1g). In cells treated with CPT and pulse-labeled with EdU, Abraxas could be detected at DNA damage sites (marked by γH2AX), which overlaps with newly synthesized DNA at replication forks (labeled by EdU), indicating that Abraxas is recruited to replication-associated DSBs (Fig. 1g).

In comparison, upon hydroxyurea (HU) treatment, the amount of ssDNA and pRPA levels were similar in Abraxas-deficient and control cells (Supplementary Fig. 1d, e), suggesting that stalled replication fork itself does not lead to increased resection in Abraxas-deficient cells. This is consistent with our previous findings that Abraxas does not play a role in responding to replication stress induced by HU at stalled replication forks for fork protection or restart of stalled replication forks^30^. Treatment with Mitomycin C (MMC) or poly (ADP-ribose) polymerase inhibitor, which forms DNA crosslink or protein–DNA crosslink that impedes DNA replication, however, led to elevated pRPA levels in *Abraxas* KO cells when compared to the control (Supplementary Fig. 1f, g). When treated with ionizing radiation (IR), compared to the control, *Abraxas* KO cells did not show elevated pRPA levels until a later time point, at 4 h after treatment, suggesting that Abraxas does not regulate the immediate processing of IR-induced two-ended DSB DNA ends, but involved in regulating resection at later steps during the repair of IR-induced DSBs (Fig. 1h). Interestingly, the increased pRPA levels in *Abraxas* KO cells at the later time point (4 h) after IR treatment also depend on replication, as addition of APH to cells 15 min before the treatment and during the incubation decreased the elevated level of pRPA (Fig. 1i).

### Abraxas suppresses R-loop accumulation and R-loop-associated DNA end resection

**Fig. 2:**
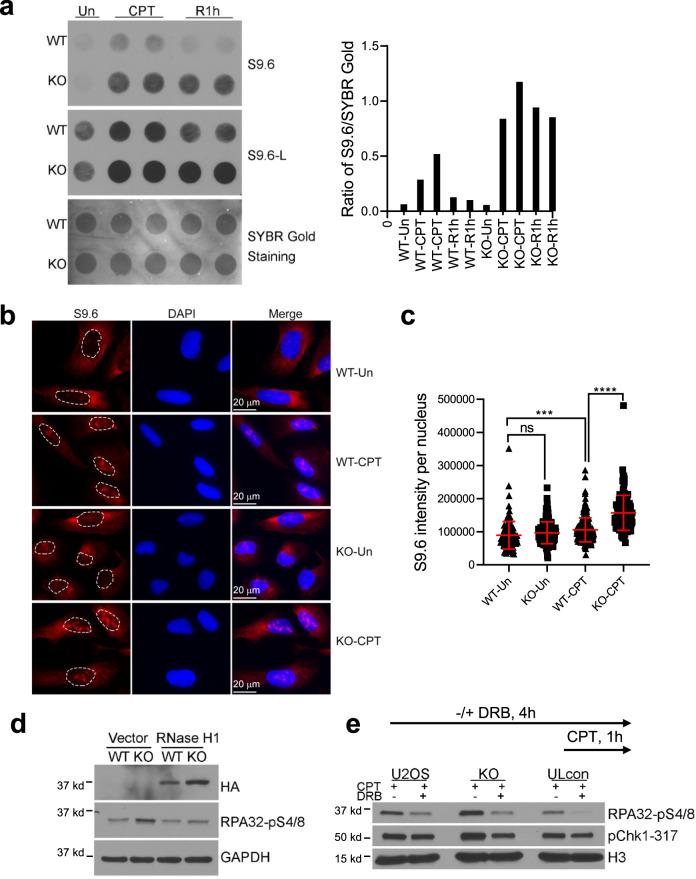
Abraxas suppresses R-loop accumulation and R-loop-associated DNA end resection. **a** Increased R loops in *Abraxas* KO cells upon CPT treatment. Dot blot using S9.6 antibody of DNA from cells untreated, treated with CPT (1 μM, 1 h), or at 1 h after release into fresh medium (left panel). Total genomic DNA was extracted and used for R-loop detection. SYBR Gold staining of the same membrane served as a loading control. S9.6 intensity was measured by ImageJ and normalized to loading control for quantification (right panel). **b** S9.6 immunofluorescence staining in WT and *Abraxas* KO cells. Cells untreated or treated with CPT (1 μM, 1 h) were pre-extracted with 0.2 % Triton X-100 before fixation. **c** S9.6 nuclear intensity was quantified by ImageJ software and plotted as mean ± SD for WT-Un (*n* = 117), KO-Un (*n* = 133), WT-CPT (*n* = 194), and KO-CPT (*n* = 121) cells examined. One-way ANOVA Kruskal–Wallis test was used for statistical analysis. ***p = 0.0002, ****p < 0.0001. **d** RNaseH1 expression reduced RPA32-pS4/8 levels in *Abraxas* KO cells treated with CPT. Cells transfected with vector or HA-tagged RNaseH1 were treated with CPT. RNaseH1 expression was detected by HA antibody. **e** Addition of transcription inhibitor DRB reduced RPA32-pS4/8 levels in *Abraxas* KO cells. Then, 20 μM 5, 6-dichloro-1-β-d-ribofuranosylbenzimidazole (DRB) was added 3 h before CPT treatment (1 μM, 1 h). See also Supplementary Fig. 2.

As TOP1 plays important roles in transcription initiation and elongation, persistent TOP1ccs can induce transcription block and formation of R-loop, a transcriptional intermediate structure with RNA : DNA hybrid and displaced ssDNA^5–7^. We tested CPT-induced R-loop formation in Abraxas-deficient cells. Using S9.6 antibody to detect R-loop levels in a dot blot assay, we found that, compared to the control, *Abraxas* KO cells showed a greater increase in R-loop levels with CPT treatment and at 1 h after release from CPT (Fig. 2a). IF staining also revealed that S9.6 nuclear signal significantly increased in *Abraxas* KO cells in response to CPT treatment (Fig. 2b, c). These data indicate that Abraxas deficiency leads to R-loop accumulation in response to CPT treatment. To determine whether R-loop formation contributes to increased resection observed in Abraxas-deficient cells, we overexpressed RNaseH1, which degrades the RNA hybridized with DNA in the R-loop structure, in *Abraxas* KO cells^36^. Overexpression of RNaseH1 reduced the elevated pRPA upon CPT treatment to the control level (Fig. 2d). It suggests that the increased resection marked by elevated pRPA in Abraxas-deficient cells is associated with R-loop processing upon treatment of CPT.

Inhibition of transcription using transcription inhibitor 5, 6-dichloro-1-β-d-ribofuranosylbenzimidazole (DRB) also decreased the elevated pRPA, suggesting that Abraxas plays a role in inhibiting resection at places when transcription machinery clashed into TOP1cc sites (Fig. 2e). Abraxas deficiency or treatment of DRB did not have much effect on cell cycle distribution, suggesting that the effect of DRB on resection is not due to a change of cell cycle distribution (Supplementary Fig. 2a). Another transcription inhibitor α-amanitin treatment was also able to reduce pRPA levels in *Abraxas* KO cells after CPT treatment (Supplementary Fig. 2b).

Together, Abraxas suppresses CPT-induced R-loop accumulation and DNA end resection at trapped TOP1cc sites associated with interference of transcriptional machineries.

### MUS81 overloading on chromatin in Abraxas-deficient cells leads to increased resection

**Fig. 3:**
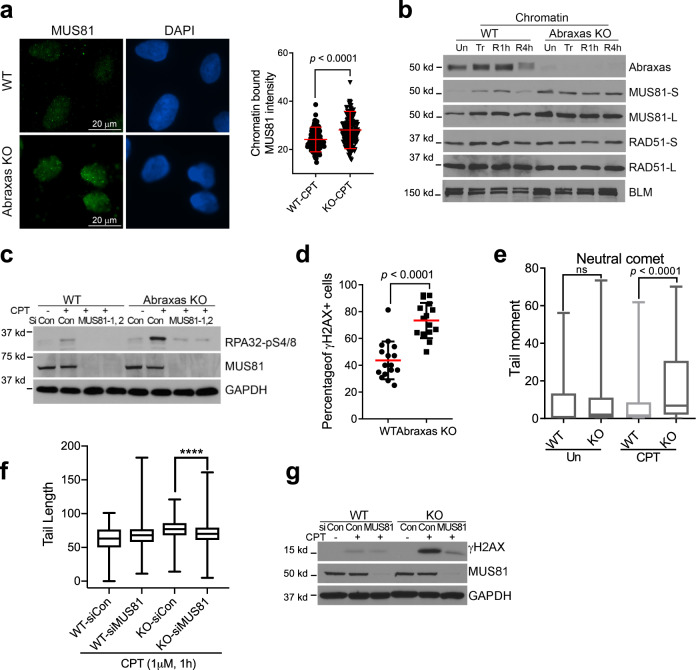
MUS81 overloading on chromatin in Abraxas-deficient cells leads to increased resection. **a** Increased chromatin-bound MUS81 in Abraxas KO cells detected by immunofluorescence. Cells upon CPT treatment were pre-extracted with 0.2% Triton X-100 before fixation and stained with MUS81 antibody. Representative images of MUS81 staining (left panel) and quantification of MUS81 nuclear intensity measured by ImageJ (right panel) are shown as mean value ± SD for WT-CPT (*n* = 142), KO-CPT (*n* = 168) cells examined. Two-tailed Student’s *t*-test was used for statistical analysis. **b** Increased chromatin-bound MUS81 in Abraxas KO cells detected by cell fractionation and western blotting. Chromatin fraction of cells untreated (Un), treated with 1 μM CPT for 1 h (Tr), or released into fresh medium for 1 h (R1h) or 4 h (R4h) after treatment were prepared for western blottings. **c** Elevated RPA32-pS4/8 levels in *Abraxas* KO cells upon CPT treatment was reduced by MUS81 knockdown. Cells transfected with siRNAs were untreated or treated with 1 μM CPT for 1 h. **d** Quantification of γH2AX immunofluorescence staining in WT and *Abraxas* KO U2OS cells treated with CPT (1 μM, 1 h). Percentage of cells containing more than 10 γH2AX foci was quantified as mean value ± SD for WT (*n* = 16 independent image areas with total 319 cells) and KO (*n* = 16 independent image areas with total 401 cells). Two-tailed *t*-test was used for statistical analysis. **e** Increased DSBs in *Abraxas* KO cells treated with CPT (1 μM, 1 h) as detected by neutral comet assay. Tail moments were measured by open comet software and plotted as box plot. Center line indicates median, box bounds indicate first and third quartiles, whiskers indicated maximum and minimum for WT-Un (*n* = 274), KO-Un (*n* = 152), WT-CPT (*n* = 383), and KO-CPT (*n* = 249) cells examined. One-way ANOVA Kruskal–Wallis test was used for statistical analysis. **f** MUS81 knockdown reduced DSBs accumulation in *Abraxas* KO cells upon CPT treatment as detected by neutral comet assay. Tail length was measured by open comet software and plotted as box plot. Center line indicates median, box bounds indicate first and third quartiles, whiskers indicated maximum and minimum for WT-*siCon* (*n* = 161), WT-*siMUS81* (*n* = 271), KO-*siCon* (*n* = 180), and KO-*siMUS81* (*n* = 286) cells examined. One-way ANOVA Kruskal–Wallis test was used for statistical analysis. ****p < 0.0001. **g** MUS81 knockdown reduced γH2AX levels in *Abraxas* KO cells upon CPT treatment. Cells transfected with indicated siRNAs were untreated or treated with 1 μM CPT for 1 h. See also Supplementary Fig. 3.

The structure-specific endonuclease MUS81 is involved in the cleavage of TOP1cc-stalled replication forks and R-loop-impeded replication forks^4,12^. We found that chromatin-bound MUS81 level was higher in *Abraxas* KO cells when compared to that of the control. IF staining of chromatin-bound MUS81 for cells pre-extracted with Triton X-100 before fixation showed enriched MUS81 intensity in *Abraxas* KO cells upon CPT treatment (Fig. 3a and Supplementary Fig. 3a). Chromatin fractionation analysis also showed that MUS81 loading on chromatin were greater in *Abraxas* KO cells (Fig. 3b). Importantly, knocking down MUS81 largely reduced elevated pRPA in *Abraxas* KO cells treated with CPT, indicating that the extensive resection of CPT-induced collapsed replication forks in *Abraxas* KO cells depends on MUS81 (Fig. 3c).

We then tested whether MUS81 overloading results in uncontrolled MUS81-mediated cleavage leading to increased DSBs. Using γH2AX as a DSB marker, we found that the percentage of cells containing γH2AX foci significantly increased (Fig. 3d and Supplementary Fig. 3b) in Abraxas KO cells treated with CPT. Neutral comet assay also showed that *Abraxas* KO cells displayed increased DSBs when compared to the control upon treatment of CPT (Fig. 3e). Importantly, knocking down MUS81 decreased CPT-induced DSBs detected by neutral comet assay and γH2AX levels in *Abraxas* KO cells (Fig. 3f, g and Supplementary Fig. 3c). EdU pulse labeling and IF with γH2AX and pRPA showed that replication-associated DSBs and resection marked by damage foci with colocalization of EdU, γH2AX, and pRPA increased in *Abraxas* KO cells treated with CPT (Supplementary Fig. 3d). Thus, Abraxas deficiency results in uncontrolled MUS81-mediated cleavage, leading to increased DSBs that are subjected to subsequent resection.

### Increased resection in Abraxas-deficient cells is independent of fork reversal but requires MRE11 endonuclease, CtIP, DNA2/BLM

**Fig. 4:**
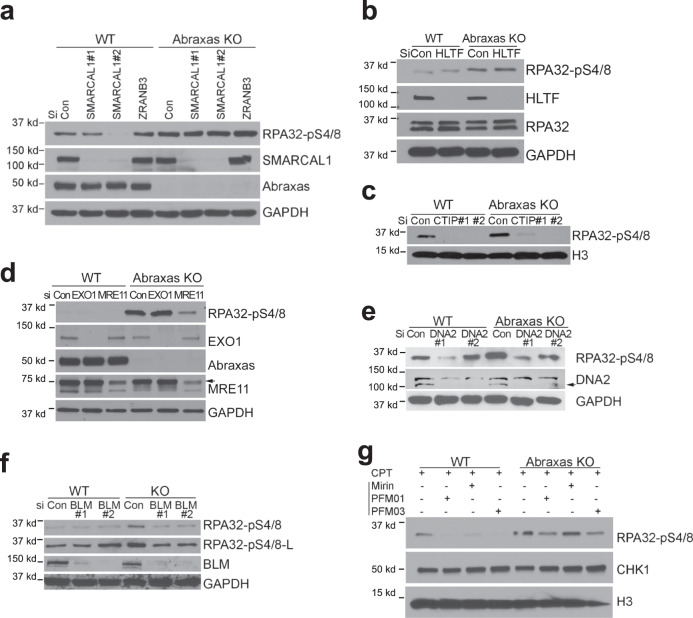
Increased resection in Abraxas-deficient cells is independent of fork reversal but requires MRE11 endonuclease, CtIP, and DNA2/BLM. **a** SMARCAL1 and ZRANB3 depletion does not rescue CPT-induced hyperphosphorylation of RPA in *Abraxas* KO cells. Data of additional siRNAs to *SMARCAL1* are in Supplementary 4a. **b** HLTF depletion does not rescue CPT-induced hyperphosphorylation of RPA in *Abraxas* KO cells. **c** Knocking down CtIP reduced the elevated RPA32-pS4/8 levels in *Abraxas* KO cells treated with CPT. CtIP knockdown efficiency is shown in Supplementary Fig. S4. **d** Knocking down MRE11 but not EXO1 reduced the elevated RPA32-pS4/8 levels in *Abraxas* KO cells treated with CPT. **e** Knocking down DNA2 reduced the elevated RPA32-pS4/8 levels in *Abraxas* KO cells treated with CPT. Data of additional siRNAs to DNA2 are in Supplementary Fig. 4a. **f** Knocking down BLM reduced the elevated RPA32-pS4/8 levels in *Abraxas* KO cells treated with CPT. **g** MRE11 endonuclease activity but not exonuclease activity is required for increased RPA32-pS4/8 levels in Abraxas KO cells upon CPT treatment. MRE11 endonuclease inhibitors PFM01 and PFM03 (100 μM) or exonuclease inhibitor Mirin (100 μM) was added 1 h before cells were treated with CPT (1 μM, 1 h). CHK1 and H3 served as loading controls. See also Supplementary Fig. 4.

MUS81 cleaves a number of DNA replication fork-like intermediates including both stalled and reversed replication fork^37,38^. To understand the effect of replication fork reversal on increased resection in Abraxas-deficient cells, we tested pRPA levels in cells depleted of SMARCAL1, ZRANB3 or HLTF, factors that are needed for replication fork reversal^39–44^. Depletion of neither SMARCAL1 nor ZRANB3 rescued hyperphosphorylation of RPA in *Abraxas* KO cells treated with CPT (Fig. 4a and Supplementary Fig. 4a, b). Depletion of HLTF also did not have much effect on the hyperphosphorylation of RPA in Abraxas KO cells (Fig. 4b). These data indicate that fork reversal is not required for RPA hyperphosphorylation and excessive end resection in Abraxas-deficient cells.

We then investigated the nucleases involved in DSBs end resection. MRE11, together with CtIP, initiates DSB end resection by generating short end resections that can be further processed by two additional nuclease complexes containing either DNA2/BLM or EXO1/BLM^13,14^. To determine whether these nucleases are involved in the excessive resection of replication-associated seDSBs in Abraxas-deficient cells, we assessed the effect of knockdown of each of these genes on pRPA levels in *Abraxas* KO cells treated with CPT. We found that knockdown of CtIP, MRE11, and DNA2/BLM, but not EXO1, reduced pRPA to the control level (Fig. 4c–f and Supplementary Fig. 4). Importantly, the cell cycle distribution was not drastically changed in *Abraxas* KO cells depleted of various nucleases, suggesting that the effect of depletion of CtIP, MRE11, or DNA2/BLM on resection was not due to a change of cell cycle distribution (Supplementary Fig. 4f). The Mre11 nuclease catalytic domain possesses both endo- and exo-nucleolytic activity, both of which are involved in end resection of two-ended DSBs. The endonuclease activity of MRE11 is required to initiate the resection and CtIP promotes the endonucleolytic activity of MRE11^45^. We further asked whether it is the endonuclease or exonuclease activity of MRE11 that is involved in the extended resection using different MRE11 inhibitors targeting the endo- or exonuclease activity^45^. Inhibitors PFM01 and PFM03, which primarily block MRE11 endonuclease activity, reduced RPA phosphorylation, whereas Mirin, an MRE11 exonuclease inhibitor, did not (Fig. 4g). These results indicate that MRE11 endonuclease activity is involved in the uncontrolled resection that occur due to Abraxas deficiency.

Together, the excessive resection of replication-associated seDSBs in Abraxas-deficient cells does not require fork reversal. It is also distinct from the resection of two-ended DSBs, requiring Mre11 endonuclease, CtIP, and DNA2/BLM, but not MRE11 exonuclease or EXO1.

### Abraxas limits K63-linked ubiquitin-dependent SLX4/MUS81 recruitment to CPT damage sites

**Fig. 5:**
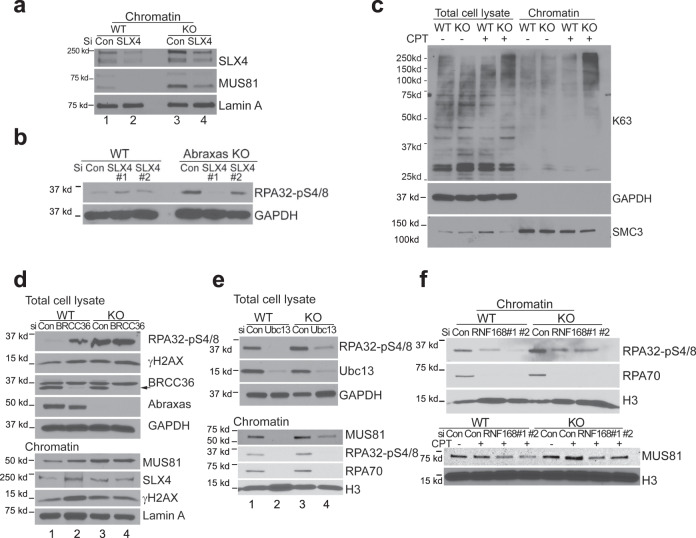
Abraxas limits K63-linked ubiquitin-dependent SLX4/MUS81 recruitment to CPT damage sites. **a** Increased chromatin-bound MUS81 in *Abraxas* KO cells was reduced by SLX4 knockdown. Cells transfected with indicated siRNAs were treated with 1 μM CPT for 1 h. Chromatin fraction was isolated for analysis by western blottings. **b** SLX4 knockdown reduced the elevated RPA32-pS4/8 levels in Abraxas KO treated with CPT. Total lysates were used for western blottings. **c** K63-linked polyubiquitination is increased in *Abraxas* KO U2OS cells treated with CPT. WT and Abraxas KO U2OS cells were untreated or treated with 1 μM CPT for 1 h. Total cell lysates and chromatin fractions were prepared from samples for western blottings. K63-linked ubiquitination was detected by antibodies against K63-linked chain. **d** BRCC36 knockdown leads to increased RPA32-pS4/8 and overloading of SLX4/MUS81 to CPT-damaged chromatin. Total cell lysates and chromatin fractions were prepared from cells transfected with indicated siRNAs and treated with CPT (1 μM, 1 h). **e** Ubc13 knockdown reduced elevated RPA32-pS4/8 levels and chromatin-bound MUS81 in *Abraxas* KO cells treated with CPT. **f** RNF168 knockdown reduced elevated RPA32-pS4/8 levels and chromatin-bound MUS81 in *Abraxas* KO cells treated with CPT. See also Supplementary Fig. 5.

The scaffold protein SLX4 interacts with MUS81 and has been shown to stimulate MUS81 activity in vitro and coordinates the resolution of Holliday junctions^46,47^. We examined whether MUS81 overloading on chromatin is dependent on SLX4. Similar to MUS81, SLX4 level was also elevated on CPT-damaged chromatin in *Abraxas* KO cells (Fig. 5a lane 1 and 3). Knocking down SLX4 significantly reduced chromatin MUS81 levels (Fig. 5a lane 3 and 4, and Supplementary Fig. 5a) and reduced pRPA in *Abraxas* KO cells (Fig. 5b and Supplementary Fig. 5b). Therefore, SLX4 functions upstream of MUS81 in promoting MUS81 chromatin loading to CPT damage sites. In contrast, knockdown of CtIP or DNA2 decreased pRPA levels in Abraxas KO cells, but had little effect on the amount of MUS81 on CPT-damaged chromatin (Supplementary Fig. 5c), consistent with the idea that these nucleases acting on resecting DSBs ends downstream of MUS81 cleavage.

SLX4 contains two tandem UBZ domains that bind to K63-linked ubiquitin chains for the recruitment of the protein to crosslink DNA damage sites^48–50^. To confirm that SLX4 recruitment to CPT damage sites through its UBZ domain is involved, we tested whether UBZ domain is important for the increased pRPA levels in Abraxas KO cells. Re-introduction of HA-tagged wild-type (WT) *SLX4* gene, but not UBZ mutant, to *Abraxas* KO cells depleted of SLX4 restored the hyperphosphorylation of RPA, indicating that the UBZ domain of SLX4 is critical in the increased resection in Abraxas KO cells (Supplementary Fig. 5d).

We then investigated whether there is an increase of K63-linked ubiquitination on CPT-damaged chromatin in Abraxas-deficient cells. We found that, compared to WT control, K63-linked ubiquitin conjugates on CPT-damaged chromatin are significantly increased in *Abraxas* KO cells (Fig. 5c and Supplementary 5e). We then examined whether depletion of BRCC36, a K63-specific DUB, leads to increased SLX4/MUS81 chromatin loading or RPA hyperphosphorylation in response to CPT. Similar to that, due to Abraxas deficiency, BRCC36 knockdown led to an increased pRPA level upon CPT treatment (Fig. 5d lane 1 and 2). Chromatin fraction analysis showed that chromatin-bound SLX4/MUS81 was also increased in BRCC36 small interfering RNA (siRNA)-treated cells (Fig. 5d lane 1 and 2). In addition, knocking down BRCC36 did not further enhance pRPA level or chromatin-bound SLX4/MUS81 in *Abraxas* KO cells upon CPT treatment, indicating that Abraxas and BRCC36 are in the same pathway in regulating DNA end resection (Fig. 5d lane 3 and 4). As Abraxas is critical for the DUB activity of BRCC36^28,29^, the elevated level of K63-conjugation in Abraxas-deficient cells is likely due to a compromised activity of BRCC36. Therefore, Abraxas restricts SLX4/MUS81 nuclease to CPT damage sites by limiting K63-linked ubiquitin conjugation on damaged chromatin.

To further confirm that uncontrolled K63-linked ubiquitin conjugation leads to increased chromatin loading of SLX4/MUS81 and excessive resection, we tested whether decreasing K63-linked ubiquitin conjugation in *Abraxas* KO cells can rescue the hyperphosphorylation of RPA in response to CPT. Knocking down the K63-specific E2 conjugating enzyme Ubc13 in KO cells decreased the amount of MUS81 on chromatin and reduced the hyperphosphorylation of RPA and chromatin-associated RPA70 levels (Fig. 5e lane 3 and 4). Reduction of RNF168, an E3 ligase involved in K63-ubiquitin conjugation, also reduced pRPA and chromatin-bound MUS81 levels in Abraxas KO cells as well (Fig. 5f and Supplementary Fig. 5f). In addition, inhibition of ATM abolished the elevated pRPA levels in Abraxas KO cells (Supplementary Fig. 5g), consistent with the previous findings that K63-linked ubiquitin modification at DSBs is dependent on ATM activation^13,26^.

Thus, through counteracting RNF168/Ubc13-dependent K63-linked ubiquitin conjugation, Abraxas/BRCA1-A complex restricts SLX4/MUS81 nuclease loading to CPT damage sites and limits excessive resection at CPT-induced DSBs.

### Abraxas limits MiDAS via RAD52, and POLD3-dependent and RAD51-independent BIR

**Fig. 6:**
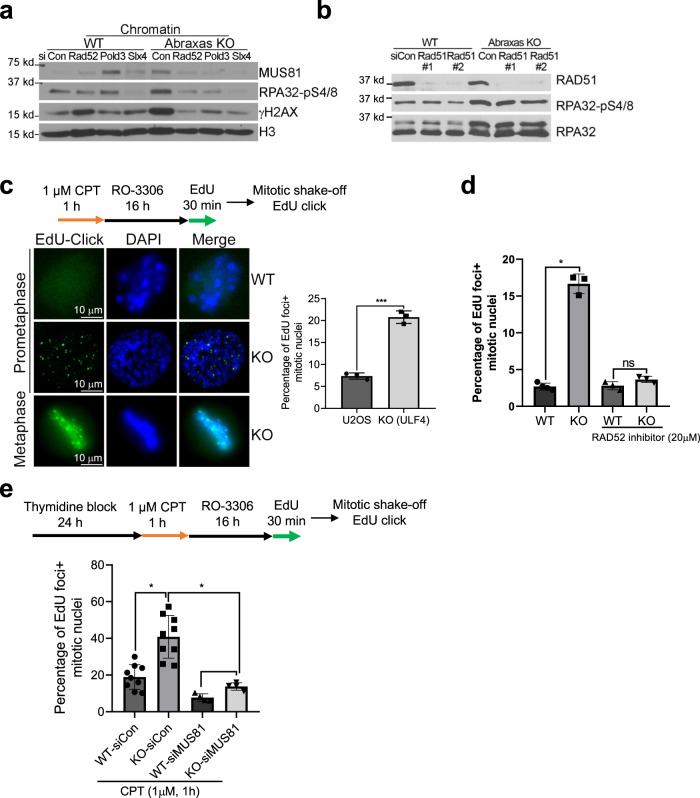
Abraxas limits mitotic DNA synthesis via RAD52, and POLD3-dependent and RAD51-independent BIR. **a** Knocking down RAD52 or POLD3 reduced RPA hyperphosphorylation and MUS81 chromatin loading in Abraxas KO cells treated with CPT. Cells transfected with indicated siRNAs were treated with CPT. Chromatin fraction were prepared and used for western blottings. **b** RPA hyperphosphorylation in *Abraxas* KO cells treated with CPT is independent of RAD51. **c** Increased mitotic DNA synthesis in *Abraxas* KO cells in response to CPT. EdU-click reaction was performed to detect mitotic DNA synthesis. A representative image of EdU foci (left panel) and quantification of the percentage of EdU foci+ cells were shown as mean value ±  SD for U2OS (*n* = 3 independent experiments with total 292 cells), KO (*n* = 3 independent experiments with total 265 cells) examined (right panel). Two-tailed unpaired Student’s *t*-test was used for statistical analysis. ***p = 0.0001. **d** Inhibition of RAD52 reduces MiDAS in Abraxas KO cells treated with CPT. Cells were treated similarly as above. RAD52 inhibitor was added during EdU pulse labeling and quantification of the percentage of EdU foci+ cells were shown as mean value ± SD for WT (*n* = 4 independent experiment with total 183 cells), KO (*n* = 3 independent experiment with total 167 cells), WT/RAD52 inhibitor (*n* = 3 independent experiments with total 121 cells), KO/RAD52 inhibitor (*n* = 3 independent experiments with total 109 cells) examined. *p = 0.042. **e** MUS81 depletion inhibits mitotic DNA synthesis in Abraxas KO cells treated with CPT. Cells transfected with indicated siRNAs were incubated with thymidine before treatment of CPT and assay for mitotic DNA synthesis. Percentage of EdU foci+ cells were quantified and shown as mean value ± SD for WT-*siCon* (*n* = 9 independent experiments with total 134 cells), KO-*siCon* (*n* = 9 independent experiments with total 108 cells), WT-*siMUS81* (*n* = 4 independent experiments with total 172 cells), and KO-*siMUS81* (*n* = 4 independent experiments with total 141 cells). One-way ANOVA Kruskal–Wallis test was used for statistical analysis. *p = 0.0440 and *p* = 0.0275. See also Supplementary Fig. 6.

Although Abraxas/BRCA1-A complex has been suggested a role in inhibiting HR in the repair of DSBs^32–34^, Abraxas deficiency led to a decrease of RAD51 foci in response to CPT (Supplementary Fig. 6a). It thus is intriguing how the increased resection in Abraxas-deficient cells effect on HR. It has been established in yeast that repair of seDSB is carried out by BIR pathway^15–17^. We thus investigated whether the excessive resection in Abraxas-deficient cells involves BIR. BIR depends on RAD52-mediated strand invasion and POLD3-dependent DNA synthesis^15–17^. We found that knocking down RAD52 and POLD3 rescued RPA hyperphosphorylation in *Abraxas* KO cells, indicating that the excessive resection in Abraxas-deficient cells is dependent on RAD52 and POLD3 (Fig. 6a and Supplementary Fig. 6). Depletion of RAD51, however, had little effect on the hyperphosphorylation of RPA (Fig. 6b). Thus, the increased resection in Abraxas KO cells likely involves BIR-like mechanism that is independent of RAD51. Interestingly, knockdown of RAD52 and POLD3 also reduced MUS81 levels on chromatin in Abraxas KO cells (Fig. 6a). This is similar to the previous finding that RAD52 promotes recruitment of MUS81 to stalled replication forks at CFSs for BIR^18^. In addition, chromatin-bound RAD52 was elevated in Abraxas KO cells treated with CPT (Supplementary Fig. 6e). It is thus likely that increased resection triggers RAD52-dependent BIR, which further promotes the recruitment of MUS81 forming a positive feedback loop that leads to even more enhanced resection.

In yeast, a majority of BIR events are completed during G2/M^17^. In mammals, MiDAS shares features with BIR for repair and restart of collapsed DNA replication forks at CFSs in response to replication stress^18,19^. We investigated whether CPT-induced increased resection in Abraxas-deficient cells is associated with MiDAS. After treatment of CPT and arrested in late G2 with a CDK1 inhibitor RO-3306, cells were released into mitosis in the presence of EdU. After collecting cells with mitotic shake-off and performing Click-it chemistry, we observed that EdU incorporation was detected in CPT-treated cells and EdU-positive mitotic cells were significantly increased in Abraxas KO cells when compared to the control (Fig. 6c). When we treated cells with RAD52 inhibitor, the increased MiDAS in *Abraxas* KO cells is abolished (Fig. 6d), indicating that the increased MiDAS in KO cells in response to CPT is dependent on RAD52, consistent with the established role of RAD52 in promoting BIR and MiDAS^18,51^. To further assess the effect of replication-associated CPT damage, we first treated cells with thymidine for 24 h arresting cells in G1/S before release into S phase for 1 h treatment with CPT and subsequent MiDAS assay (Fig. 6e). Similarly, the percentage of cells undergoing MiDAS was much greater in KO cells when compared to the control (Fig. 6e). Furthermore, knocking down MUS81 significantly reduced EdU incorporation into mitotic cells, indicating that CPT-induced MiDAS is dependent on MUS81 (Fig. 6e). This is consistent with the requirement of MUS81 for MiDAS at CFSs under replication stress^18^.

Together, these data indicate that Abraxas plays a critical role in suppressing CPT-induced damage repair through BIR mechanism such as MiDAS.

### Abraxas deficiency leads to increased chromosome aberrations and CPT sensitivity

**Fig. 7:**
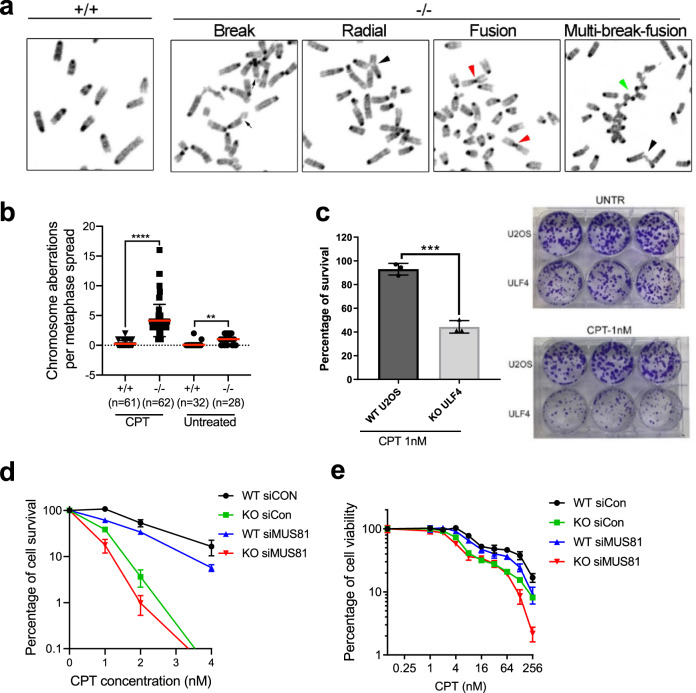
Abraxas deficiency leads to increased chromosome aberrations and cellular sensitivity to CPT. **a** *Abraxas−/−* MEFs displayed increased chromosome aberrations upon CPT treatment. Metaphase spreads were prepared from immortalized MEFs untreated or treated with 1 μM CPT for 1 h. Representative images of break (black arrows), radial structure (black arrowhead), fusion (red arrowhead), and multiple breaks and fusions (green arrowhead) of cells treated with CPT are shown. **b** Quantification of chromosome aberrations in *Abraxas−/−* and +*/+* MEFs. “*n*” represents the number of metaphases examined over three independent experiments. The red bar represents mean value and the error bars represent ±SD. One-way ANOVA Kruskal–Wallis test was used for statistical analysis. ****p < 0.0001, **p = 0.0052. **c** *Abraxas* KO U2OS cells are sensitive to CPT treatment as detected by colony formation assay. Percentage of cell survival (left) and representative images of colony formation of WT and *Abraxas* KO U2OS cells untreated or treated with CPT (right) are shown. Two-tailed unpaired Student’s *t*-test was used for statistical analysis. *N* = 3 biologically independent replicates. Data are presented as mean value ± SD. ***p = 0.0003. **d** CPT sensitivity detected by colony formation assay in WT and *Abraxas* KO U2OS cells transfected with indicated siRNA. Data are represented as mean ± SD. *N* = 3 biologically independent replicates at each CPT dose. **e** CPT sensitivity measured by cell viability assay. WT and *Abraxas* KO U2OS cells were transfected with indicated siRNA. Cells were seeded into 96-well cell culture plate at 24 h after transfection and treated with serial doses of CPT for 3 days. Viability was measured and quantified using CCK-8 kit. Data are represented as mean ± SD. *N* = 3 biologically independent replicates at each CPT dose.

BIR is a mutagenic repair pathway that is associated with elevated levels of mutagenesis and chromosomal rearrangements^15–17^. The excessive DNA end resection and increased BIR events in Abraxas-deficient cells predicts elevated genomic aberrations in Abraxas-deficient cells. We investigated chromosome aberrations in WT and *Abraxas−/−* MEF cells treated with CPT using metaphase spread. We found that chromosome aberrations significantly increased in *Abraxas−/−* MEFs, including chromosome breaks, radial structures, fusions, and multi-breaks fusions (Fig. 7a, b). *Abraxas* KO cells are sensitive to CPT as detected by colony formation assay after CPT treatment (Fig. 7c). As the excessive resection and increased MiDAS in Abraxas-deficient cells depend on MUS81, we also examined whether knockdown of MUS81 can rescue the survival of Abraxas KO cells in response to CPT treatment. We found that, however, MUS81 knockdown sensitized Abraxas KO cells to CPT as detected by colony formation assay (Fig. 7d) and cell viability assay (Fig. 7e). It is possible that although uncontrolled BIR leads to cell death, regulated BIR may still be a necessary mechanism for Abraxas-deficient cells to survive CPT-induced DNA damage. Alternatively, it is also possible that the role of MUS81 in processing other replication intermediates in restarting the collapsed replication forks is required for Abraxas KO cell survival in response to CPT.

## Discussion

**Fig. 8:**
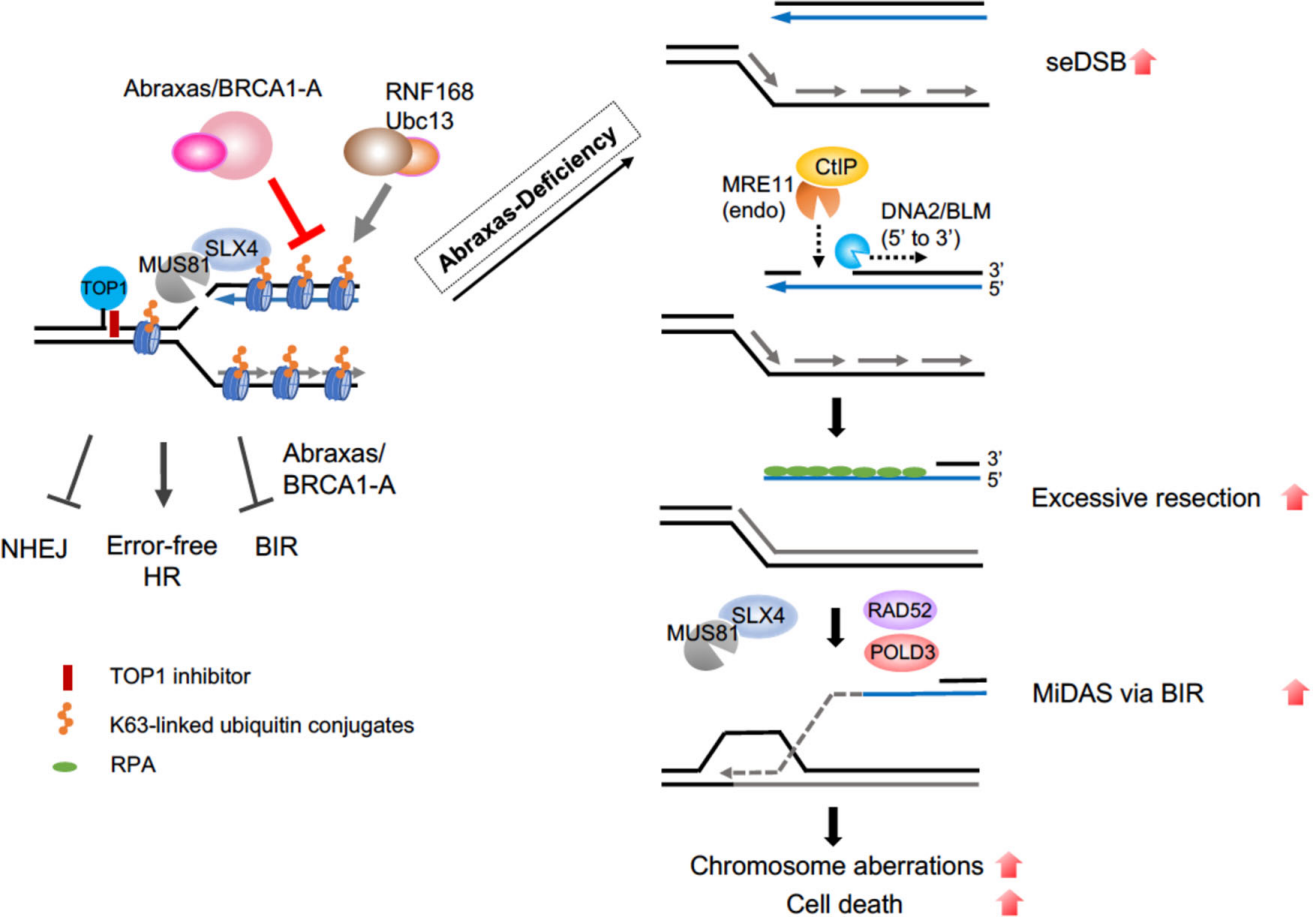
A proposed model for the role of Abraxas/BRCA1-A complex. Abraxas/BRCA1-A complex inhibits DNA end resection and limits BIR by restricting SLX4/MUS81 chromatin loading through counteracting K63-linked ubiquitin modification catalyzed by RNF168/Ubc13 in response to CPT. In the absence of Abraxas, increased K63-linked ubiquitin conjugation leads to SLX4/MUS81 overloading on chromatin, generating increased seDSBs, which are further processed by MRE11 endonucleases, CTIP and DNA2/BLM, forming an extensive length of ssDNA. Uncontrolled MUS81 loading and excessive end resection also involve RAD52- and POLD3-dependent BIR that results in increased mitotic DNA synthesis, extensive chromosome aberrations, and cell lethality in Abraxas-deficient cell.

We have demonstrated a role of Abraxas/BRCA1-A complex in limiting excessive DNA end resection, R-loop accumulation, and cells undergoing BIR-dependent MiDAS. Through counteracting RNF168/Ubc13-dependent K63-linked ubiquitin conjugation, Abraxas restricts SLX4/MUS81 recruitment to CPT damage sites for cleavage and subsequent resection to generate ssDNA. In the absence of Abraxas, increased K63-linked ubiquitin conjugation leads to SLX4/MUS81 overloading on chromatin, generating increased seDSBs, which are further processed by MRE11 endonucleases, CTIP and DNA2/BLM, forming an extensive length of ssDNA. Uncontrolled MUS81 loading and excessive end resection also involve RAD52- and POLD3-dependent BIR that results in increased MiDAS, extensive chromosome aberrations, and cell lethality in Abraxas-deficient cell (Fig. 8).

### Abraxas limits replication-associated seDSBs resection

Abraxas specifically limits end resection of replication-associated DSBs, as inhibition of DNA replication by APH completely abolished the excessive resection in Abraxas-deficient cells treated with CPT (Fig. 1). The inhibitory role of Abraxas on IR-induced DSBs end resection appears at a later time point (4 h) after treatment, possibly when neighboring replication forks emerge and collide into the DSBs, as inhibition of replication by APH significantly decreased the level of resection. MUS81 plays a central role in mediating the effect of Abraxas in inhibiting excessive DNA end resection. Abraxas inhibits DNA end resection of replication-associated DSBs by restraining K63-linked ubiquitin-dependent SLX4/MUS81 recruitment. SLX4 interacts with MUS81 and binds to K63-linked ubiquitin chain through its tandem UBZ domain modification^48–50^. Increased K63-linked ubiquitin conjugation on damaged chromatin due to Abraxas or BRCC36 deficiency leads to increased SLX4/MUS81 loading on damaged chromatin (Fig. 5 and Supplementary Fig. 5). As MUS81 mediates cleavage of CPT-induced stalled replication forks^4^, increased MUS81 chromatin loading results in elevated generation of DSBs, which are further processed by additional nucleases for DNA end resection including Mre11, CtIP, and DNA2, leading to excessive resection and generation of extended length of ssDNA in Abraxas-deficient cells. Knockdown of SLX4 or MUS81 decreases DSBs generation and eliminates the excessive resection (Figs. 3 and 5). Another piece of evidence that supports MUS81 functions upstream during the resection of DSBs ends is that knocking down downstream nucleases such as CtIP or DNA2 does not affect the amount of MUS81 on damaged chromatin but reduced excessive resection (Supplementary Fig. 5c). MUS81 is a structure-specific nuclease that cleaves replication fork-like structures in the recovery of stalled replication forks^37,38^. Collapsed replication forks induced by PARP1 trapping by olaparib, DNA crosslinks generated by MMC, or R-loop-impeded replication forks may possess similar structural features that are subjected to MUS81 cleavage and are regulated by Abraxas. The DNA structural determinant of the stalled replication intermediates of MUS81-mediated cleavage still needs to be defined but is likely to be unique, as increased resection in Abraxas-deficient cells in response to CPT is independent of SMARCAL1, ZRANB3, and HLTF, factors that are needed for replication fork reversal^39–44^.

It is noted that the excessive resection in Abraxas-deficient cells requires MRE11 endonuclease but not exonuclease activity. As CPT-induced replication-associated DSBs are seDSBs, it suggests that the initiation of resection of seDSBs is similar to that of two-ended DSBs, which is initiated by MRE11 endonuclease activity and CtIP. However, unlike the processing of two-ended DSBs, which are followed in both directions, the subsequent resection of seDSBs appears to be independent of MRE11 3′–5′ exonuclease activity such that mirin, an inhibitor of Mre11 exonuclease activity, does not decrease the resection (Fig. 4). In addition, whereas the resection is dependent on DNA2 nuclease and BLM helicase, it is independent of EXO1. For repair of seDSBs, only one broken end is available for strand invasion, which triggers BIR with DNA repair synthesis proceeding via a D-loop. It is possible that a specific complex structure arises during the progression of the resection of seDSBs that favors the use of DNA2/BLM instead of EXO1/BLM. Notably, excessive resection and increased MUS81 chromatin loading in Abraxas-deficient cells are dependent on RAD52 and POLD3, two essential factors for BIR. BIR is preceded by extensive 5′- to 3′-end resection to generate 3′-ssDNA. In addition, RAD52 appears to promote recruitment of MUS81 during replication stress-induced MiDAS at CFS via BIR^18^. Thus, it is likely that following initial MUS81-mediated cleavage, increased resection in Abraxas-deficient cells triggers the initiation of BIR and the proceeding of BIR, in turn, further re-enforces the accumulation of MUS81 and ssDNA, forming a positive feedback loop leading to MUS81 overloading, excessive end resection, and uncontrolled MiDAS.

### Role of K63-linked ubiquitin modification in replication-associated DSBs end resection

The increased recruitment of SLX4/MUS81 to chromatin in Abraxas-deficient cells is dependent on K63-linked ubiquitin modification catalyzed by RNF168 and Ubc13. As a molecular scaffold, SLX4 interacts with MUS81 and promotes MUS81 activity^46,47^. Our data indicate that SLX4/MUS81 is recruited to CPT damage sites through SLX4 UBZ domains binding to K63-linked ubiquitin chain. It is also possible that both K63-linked ubiquitin modification and replication intermediate structure formed by replication-associated DSBs are determinants of the SLX4/MUS81 recruitments.

K63-linked ubiquitin modification plays an important role in the recruitment of Abraxas/BRCA1-A complex through Rap80 binding to K63-linked ubiquitin chain. K63-linked ubiquitin modification at DSBs is dependent on ATM activation; thus, inhibition of ATM in Abraxas KO cells abolished the increased resection in response to CPT (Supplementary Fig. 5). This is consistent with the recent findings that ATM-deficiency decreases DNA end resection, and that depletion of BRCA1-A complex component in ATM-deficient cells restores DNA end resection at topotecan damaged DNA^32^. Abraxas/BRCA1-A complex, the recruitment of which depends on K63-linked ubiquitin modification, in turn, through its DUB activity, counteracts the activity of RNF168/Ubc13 in K63-linked ubiquitin conjugation, thus keeping the balance of K63-ubiquitin modification at DSBs. This balance is likely to be critical in controlling the levels of SLX4/MUS81 at DSBs. In the absence of Abraxas or BRCC36, elevated levels of K63-conjugation leads to increased chromatin loading of SLX4/MUS81 resulting in excessive end resection at replication-associated DSBs. Our study thus highlights the importance of the balance of K63-dependent ubiquitin modification at DSBs in fine-tuning the degree of resection by controlling the amount of chromatin-bound SLX4/MUS81 in response to CPT.

### Abraxas/BRCA1-A complex inhibits BIR

Decades of studies in yeast have indicated that repair of seDSBs resulted from replication fork breakage is carried out by BIR pathway^15,17^. BIR is a specific form of HR that is employed to restore the replication fork by using the sister chromatid as template. The regulation of BIR in mammalian cells, however, is less clear. RAD52- and POLD3-dependent, RAD51-independent MiDAS occurring at CFS in response to replication stress shares features with BIR and its initiation is promoted by cleavage of stalled replication forks at fragile sites by SLX4/MUS81^18,19,51^. Our studies show that, similarly, CPT-induced extensive resection that triggers MiDAS in Abraxas-deficient cells is mediated by SLX4/MUS81 cleavage and depends on RAD52 and POLD3, but not on RAD51. It is possible that the structure features associated with collapsed replication forks at CFSs leading to MiDAS are similar to those resulted by CPT-induced fork collapse, triggering RAD51-independent BIR.

BIR involves extensive DNA resection and mutagenic DNA synthesis, and is a mutagenic pathway, which when uncontrolled, can lead to cell death. How cells limit the usage of BIR in repair of DSBs is not well understood. Our studies propose that Abraxas/BRCA1-A complex plays a crucial role in restricting cells undergoing BIR in the repair of replication-associated seDSBs. The increased cellular sensitivity of Abraxas KO cells to CPT is likely due to both of the increased DSBs and the uncontrolled increase of BIR due to Abraxas deficiency. At sites of collapsed replication forks as seDSBs arise, precise resection at the break end is likely important not only to prevent NHEJ through mechanisms such as removal of Ku^52^, it is also critical in inhibiting cells undergo mutagenic HR pathway such as BIR. The K63-linked ubiquitin modification at damaged sites likely plays a crucial role in determining the precision of pathway choice and needs to be fine-tuned through balancing with the Abraxas/BRCA1-A complex DUB activity. We propose that, in addition to the choice of NHEJ and HR, a regulatory mechanism through regulating SLX4/MUS81 chromatin loading by Abraxas/BRCA1-A complex exists to control the choice of BIR and other types of HR.

Together, our study provides mechanistic insights into the role of Abraxas/BRCA1-A complex in inhibiting mutagenic repair mechanism BIR at replication-associated seDSBs to protect genome stability.

## Methods

### Cell line and cell culture

U2OS cells were grown in McCoy’s 5A with l-glutamine medium (Cellgro, Corning) supplemented with 10% fetal bovine serum (FBS) (Gibco) and 1% penicillin/streptomycin (Gibco). Abraxas KO U2OS cells were generated by using CRISPR-Cas9 as described previously^31^. 293T cell line was grown in Dulbecco’s modified Eagle’s medium (DMEM) (Cellgro, Corning) with 4.5 g/L glucose, l-glutamine, and sodium pyruvate medium supplemented with 10% FBS and 1% penicillin/streptomycin. Immortalized MEFs were cultured in DMEM with 4.5 g/L glucose, l-glutamine, and sodium pyruvate medium supplemented with 10% FBS and 1% penicillin/streptomycin.

### Chemicals, plasmids, siRNAs, and antibodies

Chemicals used in this study including CPT (Sigma, C9911), MMC (Sigma, M4287), Hydroxyurea (HU, Sigma, H8627), BrdU (Sigma, B5002), 5, 6-dichloro-1-β-d-ribofuranosylbenzimidazole (DRB, Sigma, D1916), a-Amanitin (Sigma, A2263), APH (Sigma, A0781), Thymidine (Sigma, T1895), RO-3306 (Sigma, SML0569), VE-821 (Sigma, SML1415), Mirin (Sigma, M9948), KU55933 (Selleckchem, S1092), Olaparib (Selleckchem, S1060), YOYO-1 (Invitrogen, Y3601), AICAR (RAD52 inhibitor, Sigma A9978), and PFM01 and PFM03 (from Dr. John A. Tainer).

The pENTR-RNASEH1 plasmid was purchased from MDACC shRNA and ORFeome Core in MDA Anderson and was inserted into MSCV-HA retroviral expression vector by performing Gateway Recombination Cloning Technology using LR clonase (Invitrogen). HA-tagged SLX4 WT, UBZ, and DM mutants expression plasmids were a gift from Dr. Lee Zou^50^. Expression constructs of Abraxas WT and mutants were generated previously^31^. SLX4 siRNA#1: 5′-AAACGTGAATGAAGCAGAA-3′, siRNA#2: 5′-CGGCATTTGAGTCTGCAGGTGAA-3′, and siRNA-UTR7062: 5′-GCACCAGGTTCATATGTAT-3′; MUS81 siRNA#1: 5′-CAGCCCTGGTGGATCGATA-3′ and siRNA#2: 5′-CATTAAGTGTGGGCGTCTA-3′; DNA2 siRNA#1: 5′-CAGTATCTCCTCTAGCTAG-3′, siRNA#2: 5′-ATAGCCAGTAGTATTCGAT-3′, DNA2 siRNA#3: 5′-AGACAAGGUUCCAGCGCCA-3′; BLM siRNA#1: 5′-AGCAGCGATGTGATTTGCA-3′ and siRNA#2: 5′-ATCAGCTAGAGGCGATCAA-3′; RAD51 siRNA#1: 5′-GAGCTTGACAAACTACTTC-3′ and siRNA#2: 5′-GACTGCCAGGATAAAGCTT-3′; RAD52 siRNA#1: 5′-GGCATTATGTCTAGGACTA-3′ and siRNA#2: 5′-CAATTAGTGGTTAGGGAAA-3′; SMARCAL1 siRNA#1: 5′-GCTTTGACCTTCTTAGCAA-3′, siRNA#2: 5′-AAGCAAGGCCCATCCCAAA-3′, and siRNA#3: 5′-CACCCTTTGCTAACCCAACTCATAA-3′; HLTF siRNA: 5′-GGAATATAATGTTAACGAT-3′; POLD3 siRNA: 5′-CAACAAGGCACCAGGGAAA-3′; and ZRANB3 siRNA 5′-GAGATATCATCGATTATGA-3′^12^. ON-TARGETplus human siRNA smart pool for EXO1 (L-013120-00-0005), MRE11 (L-009271-00-0005), and ON-TARGETplus Non-targeting siRNA (D-001810-01-20) were purchased from Dharmacon. BRCA1, CTIP, BRCC36, Abraxas, UBC13, and RNF8 siRNAs were purchased from Invitrogen as described previously^23,24^. siRNAs were transfected using Lipofectamine RNAiMAX (Invitrogen).

Antibodies used for immunoblotting include MUS81 (Abcam, ab14387, 1 : 1000 dilution), Abraxas (homemade, 1 : 1000 dilution)^23^, SLX4 (Bethyl Laboratories, A302-269A, 1 : 1000 dilution), HA (Cell Signaling Technology, 3724s and 2367s, 1 : 1000 dilution), K63 (EMD Millipore, 05-1308, 1 : 3000 dilution), RPA32-pS4/8 (Bethyl Laboratories, A300-245A, 1 : 2000 dilution), RPA32 (Bethyl Laboratories, A300-244A, 1 : 2000 dilution), RPA70 (Bethyl Laboratories, A300-241A, 1 : 1000 dilution), p-Chk1 S317 (Cell Signaling Technology, 2344, 1 : 500 dilution), p-Chk2 Thr68 (Cell Signaling Technology, 2661, 1 : 1000 dilution), Chk1 (Santa Cruz, sc-8408, 1 : 100 dilution), γH2AX (Millipore, 05-636, 1 : 1000 dilution), DNA2 (Abcam, ab96488, 1 : 500 dilution), BLM (Bethyl Laboratories, A300-110A, 1 : 1000 dilution), EXO1 (Millipore, ABE1354, 1 : 1000 dilution), BRCC36 (Bethyl Laboratories, IHC-00715, 1 : 1000 dilution), RAD51 (Calbiochem, PC130, 1 : 1000 dilution), RAD52 (Santa Cruz, sc-365341, 1 : 100 dilution), POLD3 (Bethyl Laboratories, A301-244A, 1 : 1000 dilution), SMARCAL1 (Santa Cruz, sc-376377, 1 : 100 dilution), HLTF (Santa Cruz, sc-398357, 1 : 100 dilution), Ubc13 (Zymed, 37-1100, 1 : 500 dilution), H3 (Abcam, ab1791, 1 : 1000 dilution), Lamin A (Sigma, L1293, 1 : 1000 dilution), CtIP (Cell Signaling Technology, 9201, 1 : 1000 dilution), RNF168 (Millipore, ABE367, 1 : 500 dilution), and GAPDH (Invitrogen, MA5-15738, 1 : 5000 dilution). Antibodies used for IF include RPA32-pS4/8 (Bethyl Laboratories, A300-245A, 1 : 1000 dilution), BrdU (BD Bioscience, 347580, 1 : 500 dilution), γH2AX (Millipore, 05-636, 1 : 1000 dilution), MUS81 (Abcam, ab14387, 1 : 200 dilution), MUS81 (Santa Cruz, sc-53382, 1 : 100 dilution), RAD51 (Calbiochem, PC130, 1 : 500 dilution), MRE11 (Novus, NB100-142, 1 : 1000 dilution), and S9.6 (kerafast, ENH001, 1 : 500 dilution).

### Immunoblotting and chromatin fractionation

Cells were lysed using NETN buffer (50 mM Tris-HCl pH 8.0, 150 mM sodium chloride, 1 mM EDTA, 0.5% Nonidet P-40, 1 mM dithiothreitol (DTT), 1 mM phenylmethylsulfonyl fluoride, 5 mM NaF, 1 mM Na_3_VO_4_, 50 mM β- glycerophosphate, and protease inhibitor cocktail), sonicated, and centrifuged at 4 °C using 21,000 × *g* for 10 min. The supernatant was collected and used as total cell lysate. For chromatin fractionation, 2 × 10^6^ cells were incubated with 300 µl cold buffer A (10 mM HEPES pH 7.9, 10 mM KCl, 1.5 mM MgCl_2_, 0.34 M sucrose, 10% glycerol, 5 mM NaF, 1 mM Na_3_VO_4_, 1 mM DTT, protease cocktail, and 0.2% Triton X-100) for 5 min on ice. After centrifugation at 1500 × *g* at 4 °C for 5 min, cell pellets were collected and washed with cold buffer A. Cell pellets were then incubated with cold buffer B (3 mM EDTA, 0.2 mM EGTA, 1 mM DTT, and protease inhibitor cocktail) for 30 min on ice. After centrifugation, pellets were collected and washed with buffer B, then resuspended in NETN buffer. After sonication and centrifugation at 4 °C using 21,000 × *g* for 10 min, the supernatant was collected and used as chromatin fraction.

### Immunofluorescence

Cells grown on coverslips were fixed with freshly made 3% paraformaldehyde/2% sucrose solution for 10 min after CPT treatment. For chromatin-bound MUS81, RAP32-pS4/8, and S9.6 staining, cells were pre-extracted before fixation using pre-extraction buffer (10 mM PIPES pH 6.8, 100 mM sodium chloride, 300 mM sucrose, 3 mM magnesium chloride, 1 mM EGTA, 0.2% Triton X-100). After fixation, cells were permeabilized with 0.5% Triton X-100 solution for 5 min on ice, washed with phosphate-buffered saline (PBS), and stained with indicated primary antibodies diluted in 1% bovine serum albumin (BSA) at 37 °C for 1 h followed by incubation with secondary antibodies conjugated with Alexa-488 (Invitrogen, A11008, 1 : 1000 dilution) or Alexa-555 (Invitrogen, A31570, 1 : 1000 dilution) for 1 h at 37 °C. Coverslips were then washed with PBS and mounted using 4′,6-diamidino-2-phenylindole (DAPI) containing antifade solution (Invitrogen). For cells pulse-labeled with EdU (10 µM) and IF staining, cells were fixed, permeabilized, and stained with indicated primary and secondary antibodies, followed by EdU-click reaction using Click-iT EdU Alexa Fluor 488 imaging kit. Images were collected with 80i eclipse Nikon microscope using ×40 or ×63 objective using NIH Elements AR software (AR 5.10.01 64 bit software).

### Native BrdU staining

Cells were labeled with BrdU (10 μM) for 36 h^53^, washed with culture medium after labeling, and treated with CPT (1 μM, 1 h). Cells were then pre-extracted with pre-extraction buffer, fixed with 3% paraformaldehyde/2% sucrose solution, treated with cold methanol for 20 min at −20 °C, washed with PBS, and treated with cold acetone for 30 s. After blocking with 2% BSA for 1 h, staining was performed with BrdU antibody followed by Alexa-488-conjugated secondary antibody. Images were captured with 80i eclipse Nikon microscope using ×40 objective. BrdU nuclear intensity was measured by ImageJ software (ImageJ 2.0 and 64 bit Java8 from https://imagej.nih.gov).

### S9.6 Dot Blot

Genomic DNA was extracted from cells using DNeasy Blood & Tissue Kit (Qiagen, 69504) following the manufacturer’s instruction. Genomic DNA (500 μg) of each sample was blotted onto positively charged nylon membrane in duplication using dot blot apparatus. After UV crosslink at 0.12 J/cm^2^ using UV Stratalinker 1800 for twice, the membrane was blocked in 5% milk/TBST buffer (20 mM Tris, 150 mM NaCl, 0/1% Tween-20), and then incubated with S9.6 antibody, followed by incubation with secondary antibody. The membrane was then reused, stripped, washed, and stained with SYBR Gold (Thermal Fisher, S11494). Image was captured using ChemiDoc imaging system (Bio-Rad).

### Neutral comet assay

Cells were trypsinized, collected, and resuspended in PBS at a concentration of 100,000 cells/ml. Ten microliters cell suspension (10,000 cells) was mixed with 100 μl low-melting agarose gel and spotted onto frosted glass slides coated with agarose gel, solidified at 4 °C in the dark. Slides were immersed into pre-chilled lysis buffer (4250-050-01, Trevigen) at 4 °C in the dark for 1 h, to lyse the cells embedded in agarose gel. After cell lysis, slides were used for electrophoresis in neural electrophoresis (Trevigen) buffer at 4 °C in the dark with a voltage at 0.6 volt/cm. Slides were fixed in 70% ethanol, stained with SYBR Gold, and imaged with 80i eclipse Nikon microscope using ×10 objective. Images were analyzed by OpenComet software (OpenComet v1.3.1 from https://cometbio.org).

### Metaphase spread

Immortalized MEF cells at 60–70% confluency were treated with 1 μM CPT for 1 h followed by incubation in medium containing 0.1 μg/ml colcemid (15210040) for 3 h. Cells were then trypsinized, collected, and incubated in 0.075 M KCl hypotonic solution, then fixed with 3 : 1 methanol : acetic acid fixative. Metaphase spreads were dropped and dried on microscopy cover slides, and stained with DAPI. Images were captured by Nikon confocal microscopy.

### MiDAS labeling and detection

MiDAS labeling and detection is carried out following procedures described^19,54^. Cells were seeded the night before to reach 80% confluency at the time when they were treated with 1 μM CPT for 1 h. Cells were then washed with fresh medium and incubated in medium with an addition of RO-3306 (9 μM) for 16 h. Cells were then washed with warm PBS and incubated in EdU (20 μM)-containing medium for 30 min for labeling. Mitotic cells were collected by shake-off, washed, and re-seeded to poly-lysine-coated coverslips. Cells were then fixed with 3% paraformaldehyde/2% sucrose solution and permeabilized with 0.5% Triton X-100 solution. EdU-click reaction was performed using Click-iT EdU Alexa Fluor 488 imaging kit (C10377) following the manufacturer’s instruction. Images were captured by Nikon confocal microscope. For thymidine synchronization, cells with 60–70% confluency were treated with 2 mM thymidine for 24 h. After wash, cells were treated with 1 μM CPT for 1 h, washed with warm PBS (37 °C) for three times, then released into RO-3306 (9 μM)-containing medium for 16 h, followed by EdU labeling and Click-iT chemistry.

### Colony survival assay

Cells were transfected with indicated siRNAs. Forty-eight hours after transfection, cells were plated at low density and incubated with indicated doses of CPT, or untreated, at 37 °C, 5% CO_2_ incubator for about 10–14 days. Colonies were fixed and stained with 0.5% crystal violet/20% methanol solution. Colony that has more than 50 cells was counted as a positive. Colony formation efficiency was normalized to untreated samples for the calculation of percentage cell survival. Error bars represent SD across triplicates.

### Cell viability assay

Cells were transfected with siRNAs and seeded at a density of 2000 cells per well into 96-well cell culture plate at 48 h after transfection. Cells were than treated with different doses of CPT continuously or left untreated for 3 days. After CPT treatment, cell viability was quantified using CCK-8 kit according to the manufacturer’s instruction (Dojindo, CK04-13) and normalization to samples without treatment.

### SMART assay

SMART assay was performed as described^35^. Cells were incubated with BrdU (10 µM) for 24 h before treatment with CPT. DNA fiber stretching in silanized coverslip was performed using Fiber Comb® molecular combing system (Genomic Vision). Coverslips containing DNA fiber were used for YOYO-1 staining or native BrdU staining. Images were captured using Nikon AI confocal microscope and analyzed using ImageJ software (ImageJ 2.0 and 64 bit Java8 from https://imagej.nih.gov).

### Real-time PCR

Total RNA was extracted using RNeasy RNA extraction kit (Qiagen) and reverse transcribed using iScript cDNA synthesis kit (Bio-Rad). Real-time PCR was performed by using SYBR green supermix (Bio-Rad) and CFX-96 real-time PCR detection system (Bio-Rad). The primers used are as follows: ZRANB3 5′-TCCCAGAGCTAAGTCCAGAAG-3′ and 5′-GCATCTGCGGTTAAGAGACCATA-3′; PPIA 5′-CAGACAAGGTCCCAAAGACAG-3′ and 5′-TCACCACCCTGACACATAAAC-3′. PPIA is used as an internal control. Primers used are listed in Supplementary Table 1.

### Statistics and reproducibility

All data were plotted as mean value with variances as SD using GraphPad Prism 8 software. Two-tailed Student’s *t*-test was used for statistical analysis of comparison of two samples. One-way analysis of variance test was used for statistical analysis of comparisons of multiple groups. All experiments were repeated at least three times. Similar results were obtained.

### Reporting summary

Further information on research design is available in the Nature Research Reporting Summary linked to this article.

## Supplementary information

Supplementary Information

Reporting Summary

## Data Availability

All relevant data are available from the authors upon reasonable request. Source data are provided with this paper.

## Source data

Source Data
