# Supplementary material for: Abraxas suppresses DNA end resection and limits break-induced replication by controlling SLX4/MUS81 chromatin loading in response to TOP1 inhibitor-induced DNA damage
Source: Nat Commun. 2021 Jul 16;12:4373. doi: 10.1038/s41467-021-24665-w (PMC8285526; doi:10.1038/s41467-021-24665-w)
Supplement: Supplementary file 1 — Supplementary Information [file 41467_2021_24665_MOESM1_ESM.pdf]

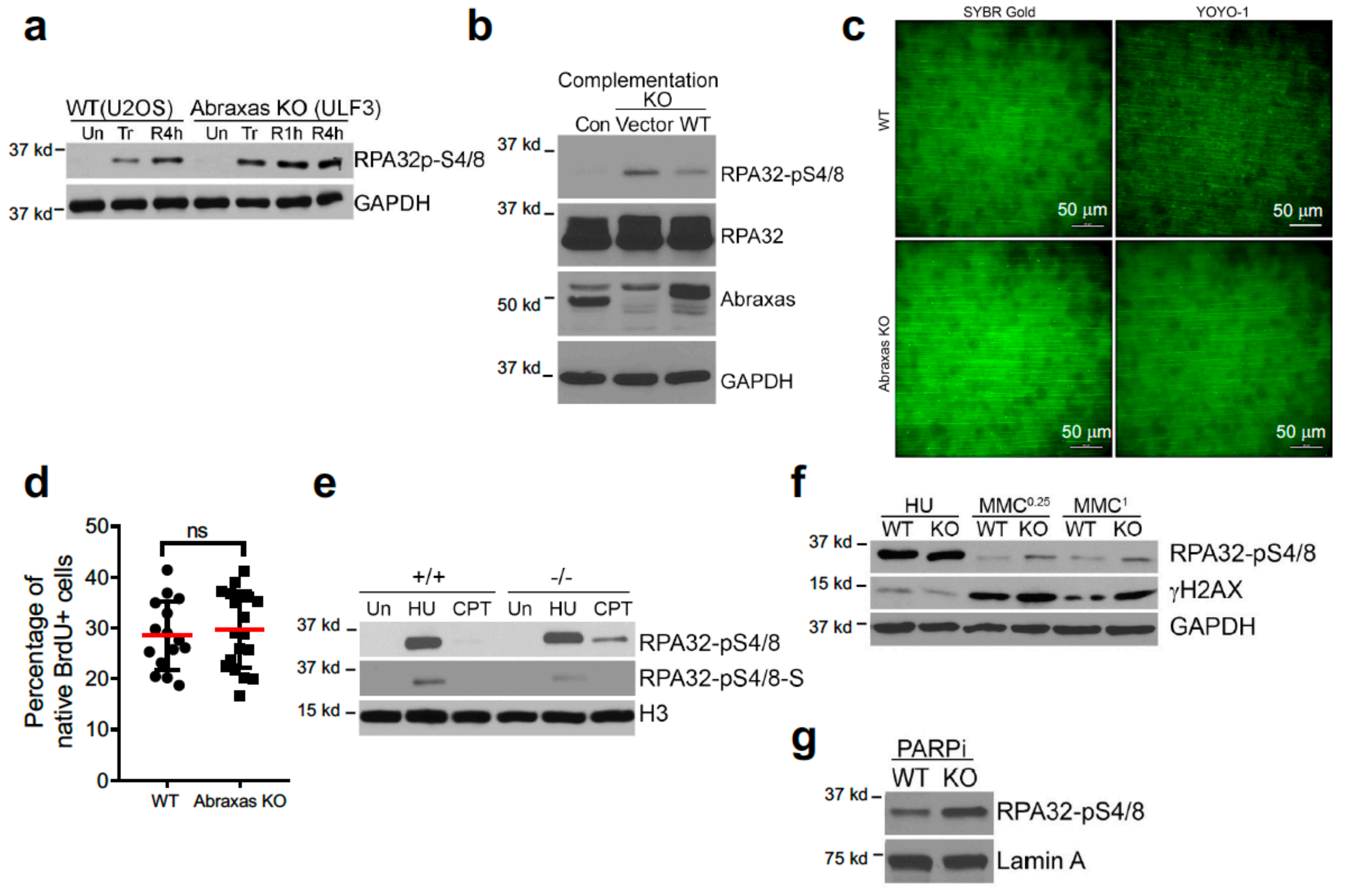

Supplementary Fig. 1. Abraxas limits DNA end resection of replication associated DSBs

(**a**) Increased RPA32-pS4/8 levels in an additional *Abraxas* KO clone (ULF3) generated by CRISPR-Cas9 in response to CPT treatment (1 μM, 1h).

(**b**) Complementation with expression of HA-Tagged Abraxas in *Abraxas* KO cells reduced hyperphosphorylation of RPA.

(**c**) Total DNA fibers (shown in Fig. 1e) in the SMART assay were visualized with staining of SYBR-gold or YOYO-1.

(**d**) Native BrdU staining of WT and Abraxas KO cells treated with HU. Cells were incubated with BrdU for 36 h before HU treatment (4mM, 4h). Immunostaining of BrdU was performed under native condition. Percentage of BrdU+ cells was plotted and shown as mean value +/- SD for WT (n=15 independent image areas with total 500 cells), KO (n=19 independent image areas with total 500 cells). Two-tailed unpaired Student's *t*-test was used for statistical analysis.

(**e**) RPA32-pS4/8 levels in Abraxas +/+ and -/- MEF cells treated with 1 μM CPT for 1 h, or 4 mM HU for 4 h.

(**f**) Increased RPA32-pS4/8 and γH2AX levels in *Abraxas* KO U2OS cells in response to MMC. Cells were treated with 0.25 mM or 1 mM MMC for 1 h, then released into fresh medium for 24 h, or treated 4 mM HU for 4 h.

(**g**) Increased RPA32-pS4/8 level in *Abraxas* KO U2OS cells upon PARP inhibitor treatment. Cells were treated with 10 μM olaparib for 24 h.

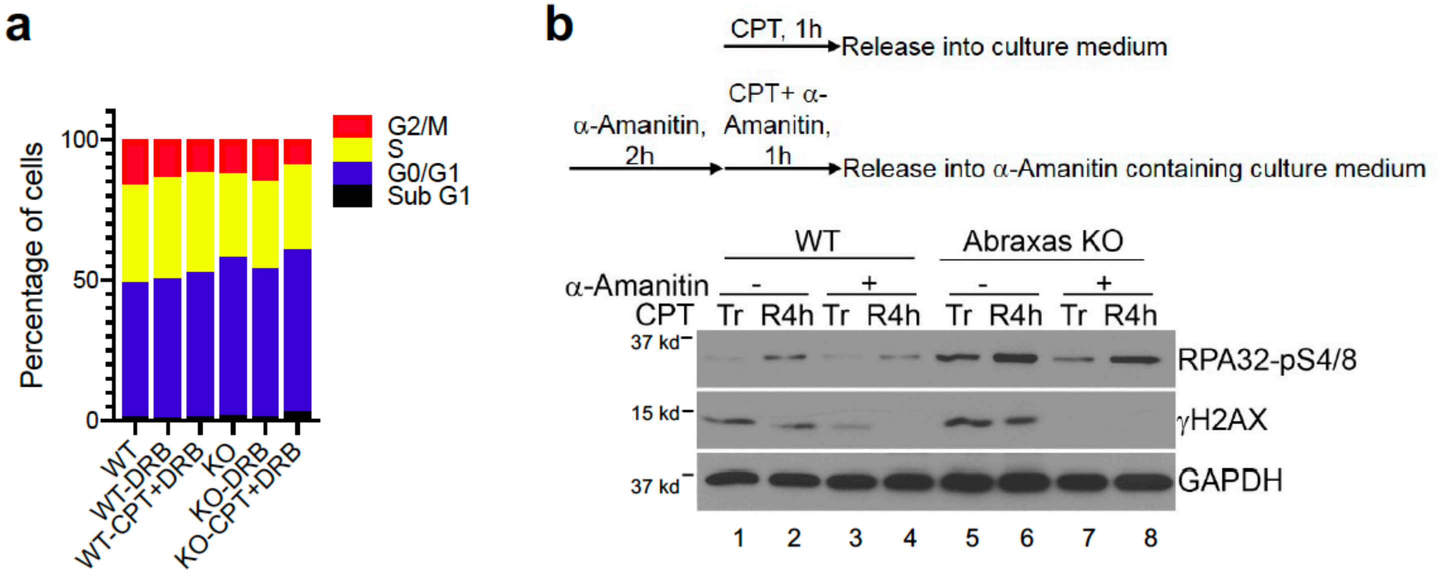

Supplementary Fig. 2. Transcription inhibitor, α-Amanitin, reduced RPA32-pS4/8 levels in *Abraxas* KO cells upon CPT treatment. (a) Cell cycle analyses of cells treated with CPT and DRB. **(b)** Amanitin was added 2 h before CPT treatment (1 μM, 1h). Cells were harvested immediately after CPT treatment (Tr) or 4 h after release (R4).

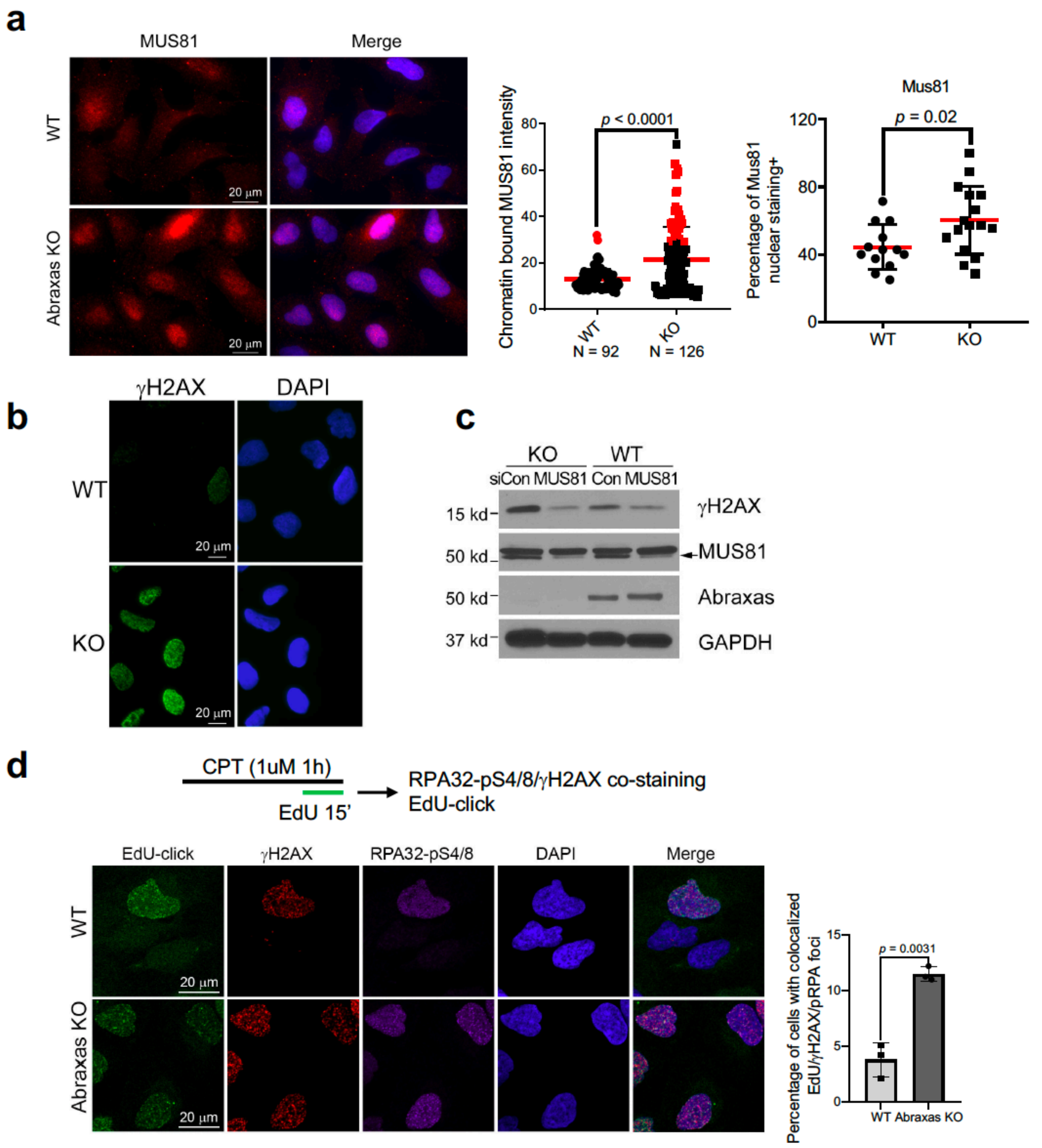

Supplementary Fig. 3. MUS81 overloading on chromatin in Abraxas-deficient cells leads to increased resection

(**a**) Increased chromatin-bound MUS81 in *Abraxas* KO cells detected by immunofluorescence using an independent MUS81 antibody (Santa Cruz, sc-53382). Cells upon CPT treatment were pre-extracted with 0.2 % triton X-100 before fixation and stained with MUS81 antibody. Representative images of MUS81 staining of WT and Abraxas KO U2OS cells (left panel) and quantification of MUS81 nuclear intensity was measured by

Image J and shown as mean value +/- SD for WT (n=92) and KO (n=126) cells examined. The percentage of cells containing MUS81+ staining (higher than the cutoff value as shown in Red color) (right panel) were quantified and shown as mean value +/- SD for WT (n = 13 independent image areas with total 102 cells), KO (n = 16 independent image areas with total 126 cells) examined. Two-tailed unpaired Student's *t*-test was used for statistical analysis.

(**b**) Representative images of γH2AX immunofluorescence staining of WT and *Abraxas* KO cells upon treatment of 1 μM CPT for 1 h.

(**c**) MUS81 knockdown reduced γH2AX levels in *Abraxas* KO cells treated with CPT.

(**d**) Increased replication associated DSBs and pRPA in *Abraxas* KO cells. Cells were treated with CPT and pulsed-labelled with EdU. Quantification data are presented as mean value +/- SD for WT (n=3 independent experiment with total 176 cells), KO (n=3 independent experiments with total 294 cells). Two-tailed unpaired Student's *t*-test was used for statistical analysis.

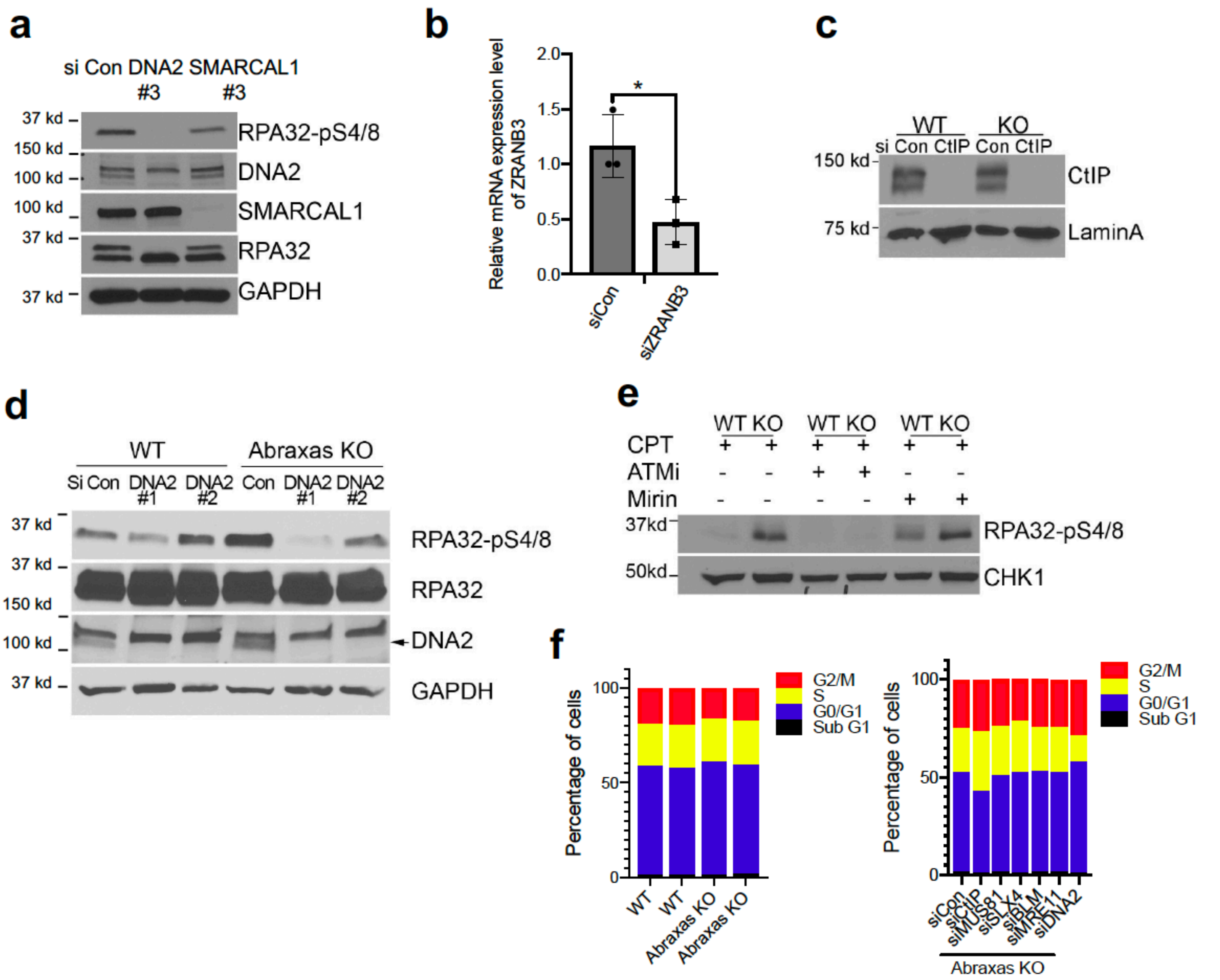

Supplementary Fig. 4. Increased resection in Abraxas-deficient cells is independent of fork reversal but requires MRE11 endonuclease, CtIP, DNA2/BLM

(a) The effect of *DNA2* si and *SMARCAL1* si on the control U2OS cells. *DNA2* si#3 behaves similarly as #1; SMARCAL1 si#3 behaves similarly as si #1 in Fig.4.

(b) Knockdown efficiency of *ZRANB* siRNAs. qRT-PCR was performed with cells treated with *siZRANB3*. N=3 biologically independent samples. Two-tailed unpaired Student's *t*-test was used for statistical analysis. "*" p=0.02

(c) CtIP siRNA knockdown efficiency was detected by western blot.

(d) DNA2 knockdown reduced CPT-induced hyper-phosphorylation of RPA in *Abraxas* KO cells.

(e) MRE11 exonuclease inhibitor mirin does not reduce RPA32-pS4/8 levels in *Abraxas* KO cells treated with CPT.

(f) Cell cycle analysis of WT or KO cells or KO cells treated with siRNAs.

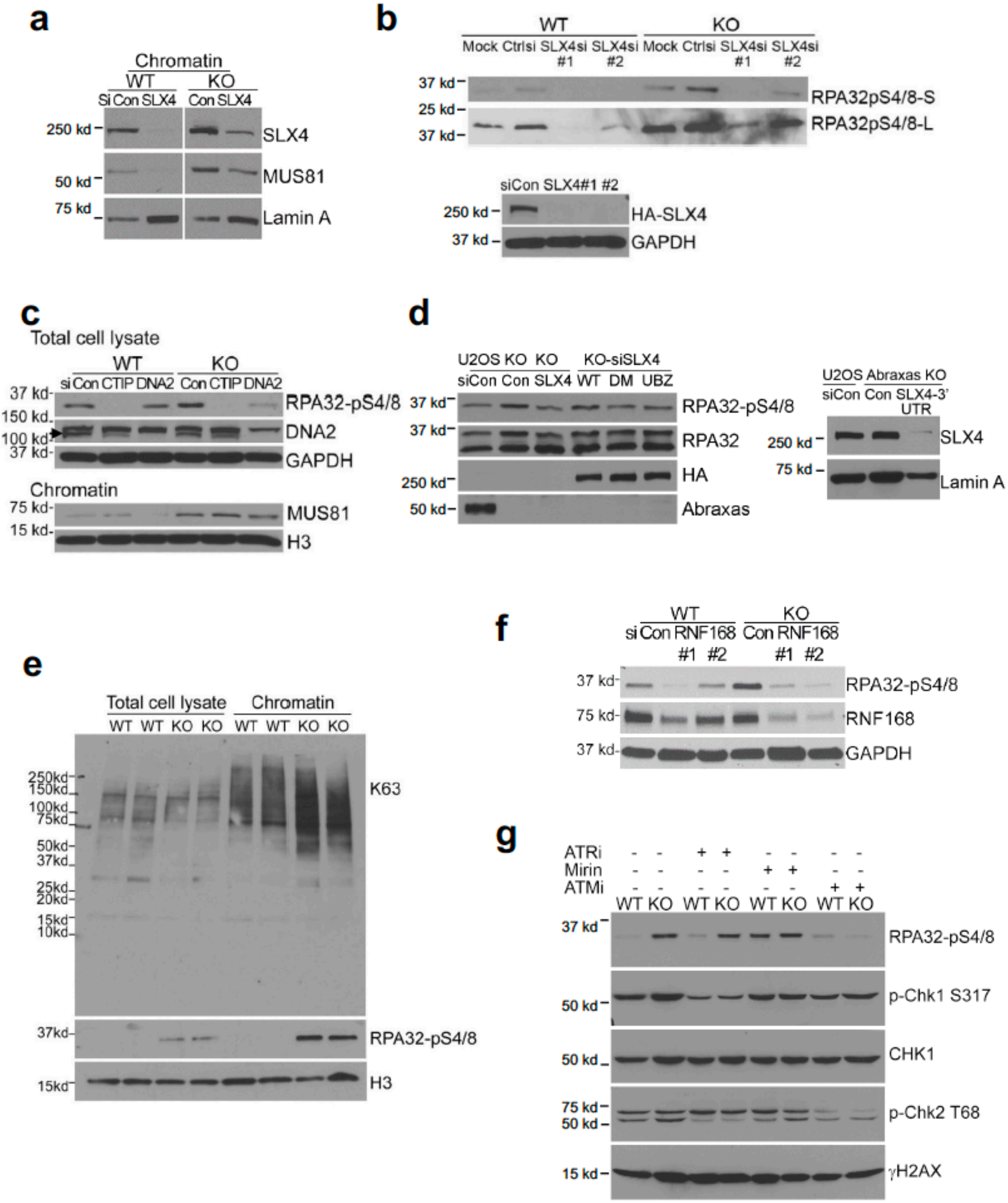

a
Chromatin
WT
KO
Si Con SLX4
Con SLX4
250 kd
50 kd
75 kd
SLX4
MUS81
Lamin A
b
WT
KO
Mock Ctrlsi SLX4si #1 SLX4si #2 Mock Ctrlsi SLX4si #1 SLX4si #2
37 kd
25 kd
37 kd
RPA32pS4/8-S
RPA32pS4/8-L
siCon SLX4#1 #2
250 kd
37 kd
HA-SLX4
GAPDH
c
Total cell lysate
WT
KO
si Con CTIP DNA2 Con CTIP DNA2
37 kd
150 kd
100 kd
37 kd
RPA32-pS4/8
DNA2
GAPDH
Chromatin
75 kd
15 kd
MUS81
H3
d
U2OS KO KO
siCon Con SLX4
KO-siSLX4
WT DM UBZ
37 kd
37 kd
250 kd
50 kd
RPA32-pS4/8
RPA32
HA
Abraxas
U2OS Abraxas KO
siCon Con SLX4-3' UTR
250 kd
75 kd
SLX4
Lamin A
e
Total cell lysate
Chromatin
WT WT KO KO WT WT KO KO
250kd
150kd
100kd
75kd
50kd
37kd
25kd
20kd
15kd
10kd
K63
37kd
RPA32-pS4/8
15kd
H3
f
WT
KO
si Con RNF168 #1 #2 Con RNF168 #1 #2
37 kd
75 kd
37 kd
RPA32-pS4/8
RNF168
GAPDH
g
ATRi
Mirin
ATMi
WT KO WT KO WT KO WT KO
37 kd
50 kd
50 kd
75 kd
50 kd
15 kd
RPA32-pS4/8
p-Chk1 S317
CHK1
p-Chk2 T68
γH2AX

Supplementary Fig. 5. Abraxas limits K63-linked ubiquitin-dependent SLX4/MUS81 recruitment to CPT damage sites

(**a**) SLX4 knockdown reduced MUS81 chromatin loading in *Abraxas* KO cells treated with CPT.

(**b**) SLX4 knockdown reduced RPA hyperphosphorylation in *Abraxas* KO cells treated with CPT. SLX4 siRNAs knockdown efficiency was assessed in cells expressing HA-SLX4 using HA antibody.

(**c**) CtIP or DNA2 knockdown does not reduce increased MUS81 chromatin loading in Abraxas KO cells treated with CPT.

(**d**) The increased pRPA in Abraxas KO cells is dependent on SLX4 UBZ domain. Abraxas KO cells were treated with SLX4 siRNAs targeting 3'UTR. KO cells depleted of SLX4 was complemented with expression of SLX4 UBZ or double UBZ and SIM (DM) mutant.

(**e**) K63-linked poly-ubiquitination is increased in *Abraxas* KO U2OS cells treated with CPT. WT and *Abraxas* KO U2OS cells were treated with 1 μM CPT for 1 h. Total cell lysates and chromatin fractions were prepared from duplicate samples for western blots. K63-linked ubiquitination was detected by antibodies against K63-linked chain.

(f) RNF168 knockdown reduced RPA hyperphosphorylation in *Abraxas* KO cells treated with CPT.

(**g**) Inhibition of ATM reduced the elevated RPA32-pS4/8 levels in *Abraxas* KO U2OS cells treated with CPT treatment. ATM inhibitor (10 μM), ART inhibitor (10 μM) or MRE11 (100 μM) inhibitor was added 15 min before CPT was used.

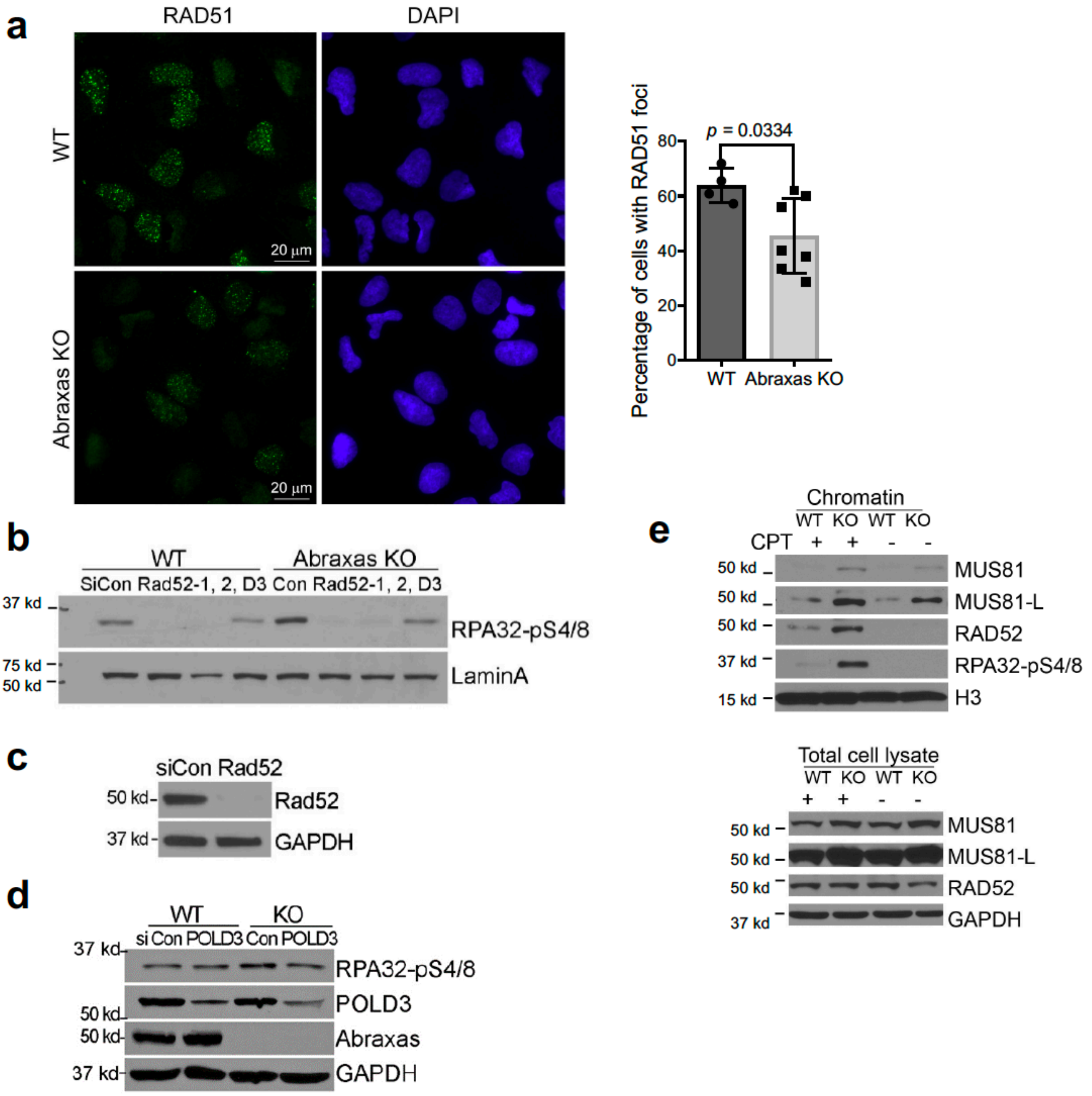

Supplementary Fig. 6. CPT-induced elevated RPA32-pS4/8 levels in *Abraxas* KO cells depend on RAD52 and POLD3 but not RAD51.

(**a**) Abraxas deficiency leads to reduced RAD51 foci in response to CPT. Quantification data are presented as mean value +/- SD for WT (n=4 independent image areas with total 169 cells), KO (n=7 independent image areas with total 132 cells). Two-tailed unpaired t test was used for statistical analysis.

(**b**) RAD52 knockdown reduced elevated RPA32-pS4/8 levels in Abraxas KO cell treated with CPT.

(**c**) RAD52 knockdown efficiency was detected by western blots.

(**d**) POLD3 knockdown reduced elevated RPA32-pS4/8 levels in Abraxas KO cell treated with CPT.

(e) Enrichment of RAD52 on CPT damaged chromatin in Abraxas KO cells.

Primers for QPCR		
Gene	Forward primer (5'-3')	Reverse primer (5'-3')
ZRANB3	TCCCAGAGCTAAGTCCAGAAG	GCATCTGCGGTTAAGAGACCATA
PPIA	CAGACAAGGTCCCAAAGACAG	TCACCACCCTGACACATAAAC

Supplementary Table 1. Primers used
