# Supplementary material for: Abraxas suppresses DNA end resection and limits break-induced replication by controlling SLX4/MUS81 chromatin loading in response to TOP1 inhibitor-induced DNA damage
Source: Nat Commun. 2021 Jul 16;12:4373. doi: 10.1038/s41467-021-24665-w (PMC8285526; doi:10.1038/s41467-021-24665-w)
Supplement: Supplementary file 2 — Reporting Summary [file 41467_2021_24665_MOESM2_ESM.pdf]

nature research

Corresponding author(s): Bin Wang
Last updated by author(s): Jun 1, 2021

Reporting Summary

Nature Research wishes to improve the reproducibility of the work that we publish. This form provides structure for consistency and transparency in reporting. For further information on Nature Research policies, see our Editorial Policies and the Editorial Policy Checklist.

Statistics

For all statistical analyses, confirm that the following items are present in the figure legend, table legend, main text, or Methods section.

n/a	Confirmed	
☐	☒	The exact sample size (*n*) for each experimental group/condition, given as a discrete number and unit of measurement
☐	☒	A statement on whether measurements were taken from distinct samples or whether the same sample was measured repeatedly
☐	☒	The statistical test(s) used AND whether they are one- or two-sided<br>*Only common tests should be described solely by name; describe more complex techniques in the Methods section.*
☒	☐	A description of all covariates tested
☒	☐	A description of any assumptions or corrections, such as tests of normality and adjustment for multiple comparisons
☐	☒	A full description of the statistical parameters including central tendency (e.g. means) or other basic estimates (e.g. regression coefficient) AND variation (e.g. standard deviation) or associated estimates of uncertainty (e.g. confidence intervals)
☐	☒	For null hypothesis testing, the test statistic (e.g. *F*, *t*, *r*) with confidence intervals, effect sizes, degrees of freedom and *P* value noted<br>*Give P values as exact values whenever suitable.*
☒	☐	For Bayesian analysis, information on the choice of priors and Markov chain Monte Carlo settings
☒	☐	For hierarchical and complex designs, identification of the appropriate level for tests and full reporting of outcomes
☒	☐	Estimates of effect sizes (e.g. Cohen's *d*, Pearson's *r*), indicating how they were calculated

Our web collection on statistics for biologists contains articles on many of the points above.

Software and code

Policy information about availability of computer code

Data collection: Immunofluorescence staining images were captured by Nikon 80i upright microscope. Metaphase spread of chromosome images were captured by Nikon confocal microscope.

Data analysis: GraphPad Prism 8 is used for statistical analysis. Unpaired student's t-test was used for comparison of two samples. One-way ANOVA Kruskal-Wallis was used for multiple comparisons. NIS Elements AR software (Nikon, AR5.10.01 64bit) and Image J (Image J 2.0 and 64bit Java8 from https://imagej.nih.gov) were used for analysis the immunofluorescence images of RPA32-pS4/8, gH2AX, BrdU, S9.6 and MUS81 staining. OpenComet (v1.3.1 from https://cometbio.org) is used for analysis of neutral comet assay images.

For manuscripts utilizing custom algorithms or software that are central to the research but not yet described in published literature, software must be made available to editors and reviewers. We strongly encourage code deposition in a community repository (e.g. GitHub). See the Nature Research guidelines for submitting code & software for further information.

Data

Policy information about availability of data

All manuscripts must include a data availability statement. This statement should provide the following information, where applicable:

- Accession codes, unique identifiers, or web links for publicly available datasets
- A list of figures that have associated raw data
- A description of any restrictions on data availability

All relevant data are available from the authors upon reasonable request. Source data are provided with this paper.

Field-specific reporting

Please select the one below that is the best fit for your research. If you are not sure, read the appropriate sections before making your selection.

☒ Life sciences ☐ Behavioural & social sciences ☐ Ecological, evolutionary & environmental sciences

For a reference copy of the document with all sections, see nature.com/documents/nr-reporting-summary-flat.pdf

Life sciences study design

All studies must disclose on these points even when the disclosure is negative.

Sample size	Sample sizes were chosen according to accepted standards in the field. Sample size was not pre-determined using statistics tolls. Minimal size of analyzed biological samples was 3. For IF staining and microscopy-based experiments, more than 100 cells per individual sample/individual experiment were counted for statistical analyses. Statistical analysis (as described in respective figure legends) was used to calculate statistical significance of obtained results. The individual p-values are indicated in figures or in figure legends.
Data exclusions	No data were excluded from the analyses.
Replication	All experiments were repeated at least three times. Similar results were obtained from each independent experiments.
Randomization	Randomization was not applicable to our study as we only compare two cell lines that are Abraxas KO versus WT cell line. For microscopy-based experiments, we randomly selected fields for image capture by using only the DAPI channel.
Blinding	Blinding was applied for cell survival assays by using ID number instead of sample names for quantification and for quantification of immunofluorescence images (without knowing the samples names but only the ID number of images). Blinding was not applied for western blot based experiments, cell cycle analyses or other experiments as all data are obtained and presented in an unbiased way.

Reporting for specific materials, systems and methods

We require information from authors about some types of materials, experimental systems and methods used in many studies. Here, indicate whether each material, system or method listed is relevant to your study. If you are not sure if a list item applies to your research, read the appropriate section before selecting a response.

Materials & experimental systems

n/a	Involved in the study	
☐	☒	Antibodies
☐	☒	Eukaryotic cell lines
☒	☐	Palaeontology and archaeology
☒	☐	Animals and other organisms
☒	☐	Human research participants
☒	☐	Clinical data
☒	☐	Dual use research of concern

Methods

n/a	Involved in the study	
☒	☐	ChIP-seq
☒	☐	Flow cytometry
☒	☐	MRI-based neuroimaging

Antibodies

Antibodies used

Primary and secondary antibodies used for immunofluoscence staining:
RPA32-pS4/8 (Bethyl Laboratories, A300-245A, 1:1000 dilution),
BrdU (BD Bioscience, 347580, 1;500 dilution),
gH2AX (Millipore, 05-636, 1:1000 dilution),
MUS81 (Abcam, ab14387, 1:200 dilution),
MUS81 (Santa Cruz, sc-53382 1:100 dilution),
S9.6 (kerafast, ENH001, 1:500 diltution),
RAD51 (Calbiochem, PC130, 1:500 dilution),
Alexa Fluor 488 goat anti-rabbit IgG (Invitrogen, A11008, 1:1000 for IF staining)
Alexa Fluor 555 donkey anti-mouse IgG (Invitrogen, A31570, 1:1000 for IF staining)

Primary antibodies used for western blot:
MUS81 (Abcam, ab14387, 1:1000 dilution),
Abraxas (Homemade, Wang et al. Science. 2007, 1:1000 dilution),
SLX4 (Bethyl Laboratories, A302-269A, 1:1000 diltuion),
HA (Cell Signaling Technology, 3724s and 2367s, 1:1000 dilution),
K63 (EMD Millipore, 05-1308, 1:3000 dilution),
RPA32-pS4/8 (Bethyl Laboratories, A300-245A, 1;2000 dilution),

RPA32 (Bethyl Laboratories, A300-244A, 1;2000 dilution),
MRE11 (Novus, NB100-142, 1:1000),
RPA70 (Bethyl Laboratories, A300-241A, 1:1000 dilution),
Chk1 (Santa Cruz, sc-8408, 1:100 dilution),
p-Chk1S317 (Cell Signaling Technology, 2344, 1:500),
gH2AX (Millipore, 05-636, 1:1000 dilution),
DNA2 (Abcam, ab96488, 1:500 dilution),
BLM (Bethyl Laboratories, A300-110A, 1:1000 dilution),
EXO1 (Millipore, ABE1354, 1:1000 dilution),
RNF8 (SantaCruz, sc-271462, 1:100 dilution),
BRCC36 (Bethyl Laboratories, IHC-00715, 1:1000 dilution),
RAD51 (Calbiochem, PC130, 1:1000 dilution),
RAD52 (Santa Cruz, sc-65341, 1:100 dilution),
POLD3 (Bethyl Laboratories, A301-244A, 1:1000 dilution),
SMARCAL1 (Santa Cruz, sc-376377, 1:100 dilution),
Ubc13 (Zymed, 37-1100, 1:500 dilution),
H3 (Abcam, ab1791, 1:1000 dilution),
Lamin A (Sigma, L1293, 1:1000 dilution),
CtIP (Cell Signaling Technology, 9201, 1:1000 dilution),
RNF168 (Millipore, ABE367, 1:500 dilution),
GAPDH (Invitrogen, MA5-15738, 1:5000 dilution)
p-Chk2 Thr68 (Cell Signaling Technology, #2661, 1:1000 dilution)
HLTF (Santa Cruz, sc-398357, 1:100 dilution)

Validation

Antibodies used for immunofluorescence staining are validated by the manufacturer or used in reference for IF experiments, and respective IF images can be found:
Rabbit anti-RPA32-pS4/8 (Figure 1b, supplemental 3d)
Mouse anti-BrdU (Figure 1c, 1e)
Mouse anti-gH2AX (Figure 1g, supplemental figure 3b, 3d)
Mouse anti-MUS81 (Figure 3a). This antibody is use for IF staining in the reference (Lemaçon D et al. MRE11 and EXO1 nucleases degrade reversed forks and elicit MUS81-dependent fork rescue in BRCA2-deficient cells. Nat Commun 8:860 (2017).
Mouse anti-MUS81 (supplemental Figure 3a). This antibody is used for IF staining in the reference (Nagaraja Chappidi et al. Fork cleavage-religation cycle and active transcription mediated replication restart after fork stalling at co-transcriptional R-Loops. Mol Cell. 2020 Feb 6; 77(3):528-541.e8.
Mouse anti-S9.6 (Figure 2b)
Rabbit anti-RAD51 (supplemental figure 6a)

Primary antibodies used for western blot are validated by the manufacturer.
Mouse anti-MUS81 (Figure 3b, 3c, 3g, 5a, 5d, 5e, 5f, 6a, supplemental figure 3c, 5a, 5c and 6e). The specificity of MUS81 antibody was further confirmed in this study by WB of siMUS81 sample shown in figure 3b and 3g.
Rabbit anti-SLX4 (Figure 5a, 5d, supplemental figure 5a, 5d). The specificity of SLX4 antibody was further confirmed in this study by WB of siSLX4 sample shown in supplemental figure 5d.
Rabbit anti-HA (Figure 2d, supplemental figure 5b and 5d).
Rabbit anti-K63 (Figure 5c and Supplemental figure 5e)
Rabbit anti-RPA32-pS4/8 (Figure 1a, 1f, 1h, 1i, 2d, 2e, 3c, 4a-4g, 5b, 5d, 5e, 5f, 6a, 6b, supplemental figure 1a, 1b, 1e, 1f, 1g, 2b, 4a, 4d, 4e, 5b, 5c, 5d, 5f, 5g, 6b, 6d and 6e)
Rabbit anti-RPA32 (Figure 1h, 6b, supplemental figure 1b, 4a, 4d and 5d)
Rabbit anti-MRE11 (Figure 4d). The specificity of MER11 antibody was further confirmed in this study by WB of siMRE11 sample shown in Figure 4d.
Rabbit anti-RPA70 (Figure 5e, and 5f)
Mouse anti-Chk1 (Figure 4g, supplemental figure 4e and 5g)
Rabbit anti-p-Chk1S317 (Figure 2e and supplemental figure 5g)
Mouse anti-gH2AX (Figure 1i, 3g, 5d, 6a, supplemental figure 1f, 2b, 3c and 5g)
Rabbit anti-DNA2 (Figure 4e, supplemental figure 4a, 4d and 5c). The specificity of DNA2 antibody was further confirmed in this study by WB of siDNA2 sample shown in Figure4e.
Rabbit anti-BLM (Figure 3b and 4f). The specificity of BLM antibody was further confirmed in this study by WB of siBLM sample shown in Figure 4f.
Rabbit anti-EXO1 (Figure 4d). The specificity of EXO1 antibody was further confirmed in this study by WB of siEXO1 sample shown in Figure 4d.
Rabbit anti-Rabbit anti-BRCC36 (Figure 5d) The specificity of BRCC36 antibody was further confirmed in this study by WB of siBRCC36 sample shown in Figure 5d.
Rabbit anti-RAD51 (Figure 3b and 6b). The specificity of RAD51 antibody was further confirmed in this study by WB of siRAD51 sample shown in Figure 6b.
Mouse amti-RAD52 (Supplemental figure 6c and 6e). The specificity of RAD52 antibody was further confirmed in this study by WB of siRAD52 sample shown in Supplemental figure 6c.
Rabbit anti-POLD3 (Supplemental figure 6d). The specificity of POLD3 antibody was further confirmed in this study by WB of siPOLD3 sample shown in Supplemental figure 6d.
Mouse anti-SMARCAL1 (Figure 4a and supplemental figure 4a). The specificity of SMARCAL1 antibody was further confirmed in this study by WB of siSMARCAL1 sample shown in Figure 4a and supplemental figure 4a.
Mouse anti-Ubc13 (Figure 5e). The specificity of Ubc13 antibody was further confirmed in this study by WB of siUbc13 sample shown in Figure 5e.
Rabbit anti-H3 (Figure 2e, 4c, 4g, 5e, 5f, 6a, supplemental figure 1e, 5c, 5e, and 6e)
Rabbit anti-Lamin A (Figure 5a, 5d, supplemental figure 1g, 4c, 5a, 5d and 6b)
Rabbit anti-CtIP (Supplemental figure 4c). The specificity of CtIP antibody was further confirmed in this study by WB of siCtIP sample shown in Supplemental figure 4c.
Rabbit anti-RNF168 (Supplemental figure 5f). The specificity of RNF168 antibody was further confirmed in this study by WB of

siRNF168 sample shown in Supplemental figure 5f.
Mouse anti-GAPDH (Figure 1a, 1f, 1h, 1i, 2d, 3c, 3g, 4a, 4b, 4d, 4e, 4f, 5b, 5c, 5d, 5e, supplemental figure 1a, 1b, 1f, 2b, 3c, 4a, 4d, 5b, 5c, 5f, 6c, 6d and 6e)
Rabbit anti-p-Chk2 Thr68 (Supplemental figure 5g)
Mouse anti-HLTF (Figure 4b). The specificity of HLTF antibody was further confirmed in this study by WB of siHLTF sample shown in Figure 4B.

Rabbit anti-Abraxas was Homemade and was validated for IF staining and WB in previous publications for our lab (Wu et al, Mol Cell, 61(3):434, 2016; Castillo et al, Cell Rep, 3(3):807, 2014). Respective IF images are shown (Figure 1g) and WB were shown (Figure 1h, 3b, 4a, 4d, 5d, supplemental figure 1b, 3c, 5d and 6d).

Dot blot analysis of R-loop was detected and showed (Figure 2a) using mouse anti-S9.6 which was validated by the manufacturer.

Eukaryotic cell lines

Policy information about cell lines

Cell line source(s)	Human U2OS and 293T cell lines were purchased from ATCC. Abraxas CRISPR-Cas9 KO U2OS cell lines were generated in our lab as previously described (Wu et al, Mol Cell, 61(3):434, 2016). Abraxas -/- and littermate +/+ mouse embryonic fibroblast (MEF) cell lines were generated in our lab as previously described (Castillo et al, Cell Rep, 3(3):807, 2014).
Authentication	U2OS and 293T cell lines were purchased from ATCC which were authenticated by ATCC. Abraxas CRISPR-Cas9 KO U2OS single clone cell lines were identified by PCR and sequencing, and by western blot (Wu et al, Mol Cell, 61(3):434, 2016). Abraxas -/- and littermate +/+ mouse embryonic fibroblast (MEF) cell lines were identified by genotyping PCR (Castillo et al, Cell Rep, 3(3):807, 2014).
Mycoplasma contamination	Cell lines were all tested negative for mycoplasma using PCR based mycoplasma detection method.
Commonly misidentified lines (See ICLAC register)	No commonly misidentified cell lines were used in this study.
